# Manganese-derived biomaterials for tumor diagnosis and therapy

**DOI:** 10.1186/s12951-024-02629-8

**Published:** 2024-06-15

**Authors:** Peiying Huang, Qinglai Tang, Mengmeng Li, Qian Yang, Yuming Zhang, Lanjie Lei, Shisheng Li

**Affiliations:** 1 Department of Otorhinolaryngology Head and Neck Surgery, The Second Xiangya Hospital, Central South University, Changsha, Hunan 410011 China; 2 Key Laboratory of Artificial Organs and Computational Medicine in Zhejiang Province, Institute of Translational Medicine, Zhejiang Shuren University, Hangzhou, Zhejiang 310015 China

**Keywords:** Manganese-derived biomaterials, Magnetic resonance imaging, Tumor microenvironment, Nanomaterials, Drug delivery

## Abstract

Manganese (Mn) is widely recognized owing to its low cost, non-toxic nature, and versatile oxidation states, leading to the emergence of various Mn-based nanomaterials with applications across diverse fields, particularly in tumor diagnosis and therapy. Systematic reviews specifically addressing the tumor diagnosis and therapy aspects of Mn-derived biomaterials are lacking. This review comprehensively explores the physicochemical characteristics and synthesis methods of Mn-derived biomaterials, emphasizing their role in tumor diagnostics, including magnetic resonance imaging, photoacoustic and photothermal imaging, ultrasound imaging, multimodal imaging, and biodetection. Moreover, the advantages of Mn-based materials in tumor treatment applications are discussed, including drug delivery, tumor microenvironment regulation, synergistic photothermal, photodynamic, and chemodynamic therapies, tumor immunotherapy, and imaging-guided therapy. The review concludes by providing insights into the current landscape and future directions for Mn-driven advancements in the field, serving as a comprehensive resource for researchers and clinicians.

## Introduction

The rising prevalence of cancer represents a major global public health burden [1, 2]. Late diagnosis and tumor metastasis are primary causes of high mortality in patients with cancer [3]. Currently, several methods are available for the early diagnosis of cancer, from traditional tumor diagnosis methods, such as imaging monitoring, magnetic resonance imaging (MRI) [4], Circulating tumor cell technology, and electrochemical sensing [5]. MRI is extensively used in clinical practice owing to its high spatial resolution, deep signal penetration, and excellent soft tissue contrast, attracting considerable attention and research efforts. However, MRI also has the disadvantages of low sensitivity and accuracy [6] Addressing these limitations, the optimization of contrast media has become a widely studied and promising method. Currently, metal-based MRI contrast agents (CAs) are commonly used clinically, including paramagnetic Gd^3+^ chelates (T1, positive CAs) and superparamagnetic Fe_3_O_4_ nanoparticles (T2, negative CAs) [7–9]. Unfortunately, Gd-based agents cause potentially lethal nephrogenic systemic fibrosis [10]. Furthermore, agents containing Fe have the capacity for heavy metal toxicity in the human body and pose a risk of allergic reactions that may result in life-threatening consequences [11, 12]. Therefore, both options pose safety risks.

Manganese (II) and its derivatives, known for their good biodegradability, biocompatibility, low toxicity, high spin numbers, and long electron relaxation times, are promising alternatives to MRI CAs. Hence, considerable attention has been devoted to the research and development of Mn-based agents over the past decade [13–15]. In 1973, Lauterbur et al. first proposed the use of MnSO_4_ as a CA in MRI for enhanced T1-weighted imaging [16]. Mn-based contrast media have been administered to tumors [17, 18]. In 2006, Elizaveta et al. introduced CMC-001, an oral contrast medium containing Mn^2+^, enhancing the visualization of liver metastases using computed tomography (CT) and MRI. To improve detection contrast and diagnostic accuracy, Chen et al. developed a novel Mn complex, Mn-BnO-TyrEDTA, as a liver-specific CA [19]. Owing to less toxicity, enriched biochemical features, and favorable electronic configuration in Mn-based CAs, several imaging agents using Mn complexes serve as MRI probes, with some Mn complexes approved as clinical MRI CAs (e.g., Teslascan™ and LumenHance™) [20, 21]. Notably, single-modality imaging techniques often fail to offer comprehensive and accurate information and are sometimes unable to meet the requirements of precision medicine. Mn-based CA possess chemically identical radioactive and paramagnetic properties, making them suitable for multimodal imaging techniques that enable both double and simultaneous detections. These agents hold promise as bimodal CAs for cancer detection.

**Fig. 1:**
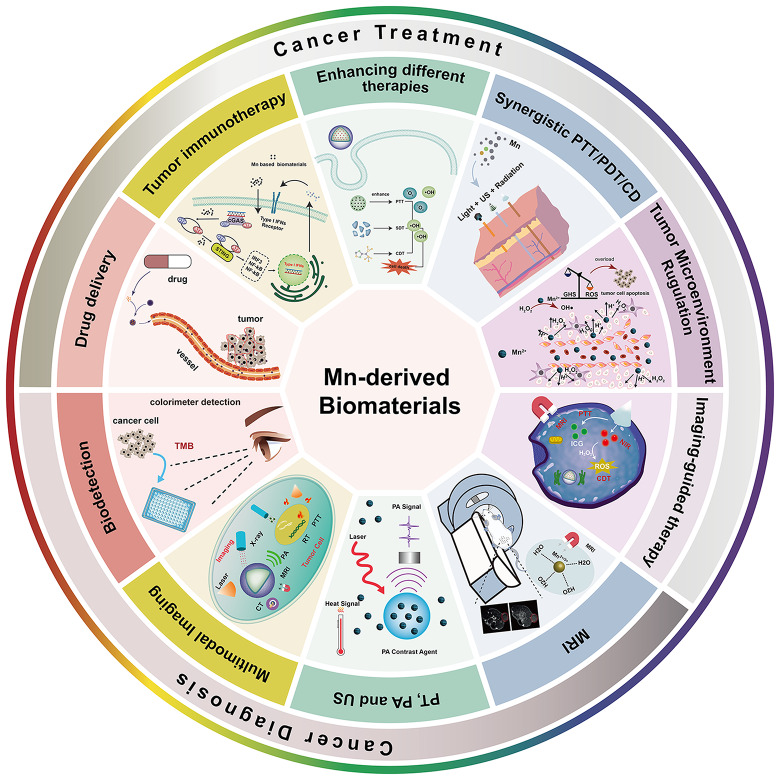
Summative scheme of manganese-derived biomaterials for tumor diagnosis and therapy

Conventional cancer treatments, including surgery, chemotherapy, and radiotherapy, have been widely employed [22, 23]. However, the inherent tumor heterogeneity and complex tumor microenvironment (TME) often hinder the effectiveness of chemotherapeutic drugs and promote metastasis. Moreover, these commonly used treatment modalities are associated with diverse adverse effects, such as tissue damage, and clinical symptoms such as fever, nausea, respiratory distress syndrome, and vomiting disorders [24]. Emerging strategies such as chemodynamic therapy (CDT), photodynamic therapy (PDT), and immunotherapy have attracted considerable attention in biomedical research [25]. Mn has received attention owing to bioimaging and anti-cancer activities. Mn-based biomaterials can release Mn ions, initiating the production of hydroxyl radicals (•OH) through reactions with hydrogen peroxide (H_2_O_2_) in the TME. This catalyzes Fenton-like reactions, generating highly reactive hydroxyl radicals for CDT, a common tumor treatment [26–28]. Additionally, the two-dimensional nanostructures of Mn-derived biomaterials, owing to their large surface area and exceptional photothermal conversion efficiency, can absorb near-infrared (NIR) light and convert it into heat, thereby effectively destroying tumor cells while minimizing the impact on surrounding healthy tissues. This capability is extensively utilized in photothermal therapy (PTT) and photoacoustic therapy (PAT), enhancing the efficacy and precision of cancer treatments. For instance, the Mn-based composite BSA-Ce6@IrO_2_/MnO_2_ has shown a photothermal conversion efficiency of up to 65.3% [29]. Combining manganese nanomaterials with other metallic components can enhance photothermal conversion efficiency. Mn-based biomaterials serve as biocompatible drug delivery systems for the controlled release of various drugs [30, 31]. For example, MnO_2_ nanosheets have the potential to serve as biocompatible drug delivery systems, enabling efficient delivery of photosensitizers (PS) into cancer cells [32]. Additionally, as an essential trace element for humans, Mn with different oxidation states plays an important role in cell metabolism and the immune system. Mn^2+^ facilitates cGAS and STING activation from the production of cGAMP to the binding affinity of cGAMP/STING, eliciting a robust antitumor immune response [33]. Mn (II) ions with high spin numbers (S = 5/2, I = 5/2), long electron relaxation times, and five unpaired electrons are among the best paramagnetic metals used in MRI CAs. However, Mn has potential applications in cancer therapy [26, 34–36]. Mn-derived biomaterials have garnered increasing interest in tumor diagnosis and therapy owing to the good biocompatibility and extraordinary functions of Mn. Despite reviews focusing on the roles of manganese-based nanomaterials in imaging diagnostics and the assistance of various cancer therapies [37–39], there is a lack of systematic reviews that comprehensively address their synthesis methods, physicochemical properties, toxicity profiles, and extensive application in tumor diagnosis and therapy. This review aims to address this gap by providing the synthesis, characterization, and application of Mn-derived biomaterials in biological and medical fields, and exploring their diverse applications in cancer diagnosis and therapy. The status of tumor diagnosis and therapy using Mn-derived biomaterials over the past two decades was reviewed (Fig. 1). Moreover, the applications of Mn-derived biomaterials and suggestions for future research have been proposed in this review, providing a reference for the development and clinical translation of Mn-based biomaterials.

## Synthesis of manganese derived biomaterials

The synthesis of manganese-derived biomaterials involves various methods to harness the full potential of both the biological activity of manganese and the physicochemical properties of biomaterial. This encompasses various methods such as precipitation, hydrothermal, micro-emulsion, sol–gel, and sonochemical technique [40–50]. The impact of these materials extends across diverse applications, including catalysis, pharmaceuticals, optics, food technology, cosmetics, water treatment, electrochemistry, energy, biomedicine, biosensors, cancer therapy, healthcare, and drug delivery. The focus is often on manganese oxide nanoparticles including manganese dioxide, dimanganese trioxide, trimanganese tetraoxide (MnO, MnO_2_, Mn_3_O_4_), etc. Different types of Mananese and Manganese-derived nanomaterials have demonstrated diverse applications in tumor diagnosis and treatment, and their preparation strategies vary. Consequently, the selection of different preparation methods is crucial for Mn nanoparticles. Depending on the synthesis procedure, precursors, and Mn source, Mn nanomaterials with different functionalities and advantages are formed. Table 1 comprehensively discusses the detailed methods for manufacturing and preparing these innovative biomaterials, emphasizing their advantages, applications, and specific approaches employed.

**Table 1:** Comparison of advantages and disadvantages of common synthesis methods of manganese-derived biomaterials

Preparation strategy	Mn sources	Summary process	Principle	Advantages	Materials	Application	Ref.
Hydro or solvothermal	Mn (II) acetate Mn (II) stearate	Dissolving raw materials in aqueous or other good solution and reacting in Teflon-lined autoclave	High-temperature and high-pressure liquid phase nonequilibrium reaction	Narrow size distribution	α- MnO_2_, β- MnO_2_, δ-MnO_2_ Mn_3_O_4_ NP_S_	T1 contrast agent Chemotherapy	[40] [41] [42, 51]
Redox method	KMnO_4_ MnCl_2_	Control of manganese valence state using diverse reducing and oxidizing agents	Redox reactions and electron transfer	Aqueous-phase synthesis large specific surface area	MnO_2_ nanosheets	Glutathione detection Drug delivery	[43] [44]
High temperature decomposition	Manganese (II)	Precursor compounds decomposited at high temperatures, resulting in the formation of nanoparticles with controlled size, shape, and composition	An oxygen-free organic- phase synthetic process	Tunable size and morphology	Core–shell MnS@Bi_2_S_3_	MR/PA/CT imaging siRNA delivery	[45]
Chemical co-precipitation method	Manganese acetate Manganese nitrate	Simultaneously precipitates multiple substances from solutions with different metal ions	Controlled reaction between cation and anion	Simple and rapid, green	MnO_2_	Image-guided nanodelivery	[46] [47]
Sol–Gel	Mn-containing organic metal alkoxides, esters, etc.	Sol–gel method mainly undergoes in few steps to deliver the final metal oxide protocols and those are hydrolysis, condensation, and drying process	Hydrolysis of the metal alkoxides and condensation of the hydrolyzed intermediates	Control the product morphology	Nanoparticles or nanozymes	Large surface-area-to-mass ratios	[48]
Microwave-assisted method	MnCl_2_•4H_2_O	The reaction mixture was irradiated in a microwave oven to accelerate chemical synthesis	Dielectric polarization leading to heating effect	Well-controlled size and magnetic properties	Manganese ferrite nanoparticles	MRI contrast agents	[49]
Green synthesis	Manganese solution	Reducing manganese ions in manganese solution using pure lemon extract	Reduction reaction	High-precision and tight control of etch variability	Manganese nanoparticles	Antibacterial biomaterials	[50]

### Hydrothermal/solvothermal

**Fig. 2:**
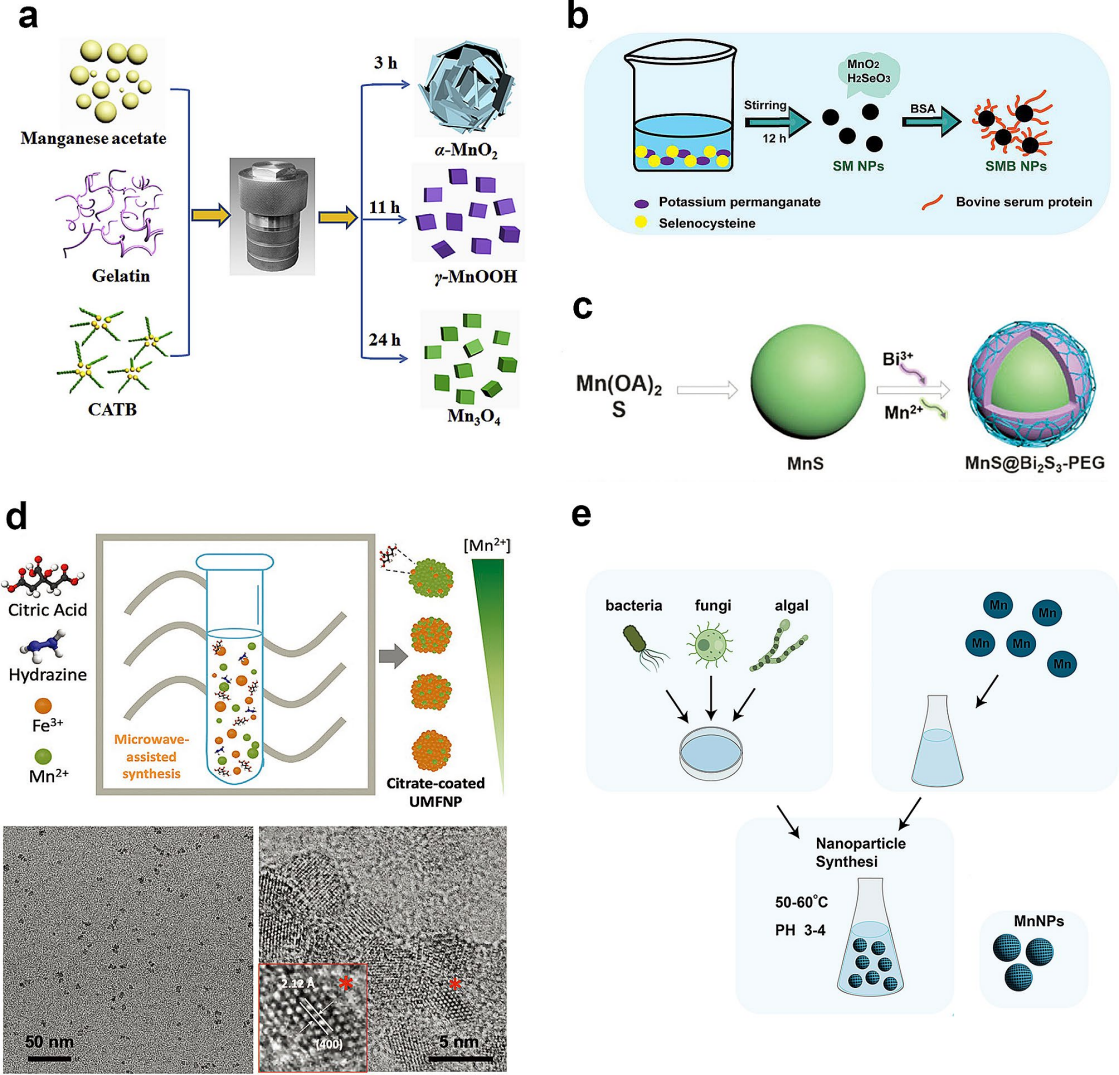
The synthesis process of Manganese-derived biomaterials. (**a**) Illustration of Hydrothermal Method on the synthesis route for α-MnO2, γ-MnOOH, and Mn_3_O_4_ nanomaterials [40] Copyright 2018, Elsevier. (**b**) Illustration of redox methods to prepared biocompatible manganese selenium-based nanoparticles [44] Copyright Yu Y, Fan P, Li J, Wang S, 2023, MDPI, Fig. 1. Reproduced under CC BY 4.0 (https://creativecommons.org/licenses/by/4.0/). (**c**) A schematic of high temperature deposition synthesis of MnS@Bi_2_S3-PEG [45] Copyright 2017, The Royal Society of Chemistry. (**d**) Illustration of the microwave-assisted method used to produce citrate-coated UMFNPs with fine control in the chemical composition [49] Copyright 2022, Wiley-VCH GmbH. (**e**) Illustration of green synthesis method for MnNPs

Hydrothermal and solvothermal are synthesis methods used to produce materials under high-temperature and high-pressure conditions. The hydrothermal/ solvothermal synthesis method involves dissolving the raw materials in water or another solvent to create an aqueous solution, which is then subjected to a reaction in a hydrothermal autoclave (Fig. 2a) [40]. Wang and Li conducted hydrothermal/solvothermal synthesis to create nanoparticles with α- and β-phases [51]. Their methodology involved adjusting the concentrations of ammonium and sulfate ions during the reaction between ammonium persulfate and manganese sulfate. This approach resulted in the successful fabrication of nanoparticles with unique structural characteristics. Furthermore, Wang and Li extended their exploration to proportional reactions of potassium permanganate and manganese sulfate under hydrothermal conditions, yielding a diverse range of MnO_2_ phases, including α-, β-, and δ-MnO_2_. Another method involves heating manganese oleate in high-boiling-point solvents such as 1-octadecene to induce nucleation and particle growth [52]. This method allows for the preparation of nanoparticles with precise size control. This innovative approach showcases the adaptability of hydrothermal/solvothermal synthesis for tailoring the structural properties of manganese oxide nanoparticles, presenting a versatile platform for the controlled synthesis of nanomaterials with distinct phases and functionalities.

### Redox method

In this method, the manipulation of the oxidation state of manganese is achieved through the use of diverse reducing and oxidizing agents. The Redox processes involve a dynamic interplay of electron transfer, leading to either the reduction or oxidation of manganese ions. Yang et al. successfully prepared biocompatible manganese selenium-based nanoparticles (SMB NPs) using a straightforward one step redox method, followed by modification with bovine serum albumin (BSA) to achieve physiologically stable SMB NPs (Fig. 2b) [44]. The dynamic interplay of electron transfer in redox processes allows for precise control over the oxidation states of the involved elements, contributing to the formation of well-defined and stable nanoparticles. This simplicity, efficiency, and versatility make it advantageous for synthesizing a variety of nanoparticles with tailored properties for specific applications.

### High temperature decomposition

High temperature decomposition is one of the most convenient techniques for the preparation of nanomaterials with high-quality monodisperse nanocrystals with pure phase and narrow particle size distributions. Li et al. prepared core-shell MnS@Bi_2_S_3_ through high temperature decomposition (Fig. 2c) [45]. The process allows for precise tuning of parameters to achieve desired characteristics, making it a versatile and effective method for nanomaterial synthesis.

### Precipitation

The technique of simultaneously precipitating multiple substances from a manganese (II) nitrate solution, influenced by pH, temperature, and concentration, is achieved through the method of co-precipitation. This process involves combining the manganese (II) nitrate solution with another solution containing different metal ions. By carefully adjusting the pH, temperature, and concentration of the solution, multiple metal ions can precipitate simultaneously to form solid products. Mn_3_O_4_ can prepared by precipitation method. Mohammed et al. detailed the preparation of Mn_3_O_4_ nanoparticles using a precipitation method [53]. A manganese nitrate solution was prepared and subjected to a controlled heating and stirring process in a reaction vessel. Sodium bicarbonate was added gradually to adjust the pH to 9. After maintaining the reaction conditions for an additional 3 h, the mixture was cooled and stirred overnight. The precipitate was then separated by centrifugation, dried, and finally calcined at high temperatures, leading to the successful synthesis of Mn_3_O_4_ nanoparticles.

### Sol-gel methods

The Sol–gel method involves the hydrolysis of metal precursors in the presence of substances like alcohol, water, acid, or base. During this process, the solution undergoes condensation, leading to the formation of a gel. Subsequently, the excess solvent is removed from the system. This technique allows for precise control over the composition and structure of the resulting material, making it a valuable approach for creating diverse substances such as thin films, coatings, and nanoparticles, with applications spanning various scientific and industrial domains. Xue et al. focused on employing the sol-gel method to synthesize Manganese-doped mesoporous silica nanoparticles (MMSN) [54]. In their experimental procedure, cetyltrimethyl ammonium bromide was introduced into an ethanol solution, with the proper pH level. The mixture was heated, then tetraethyl orthosilicate and MnCl2 were added with continuous stirring. After several processing steps including centrifugation, vacuum drying, and calcination, MMSN were synthesized. Post-synthesis, these nanoparticles, referred to as PMMSN following plasma adsorption and freeze-drying, underwent further analysis. This example underscores the effectiveness of the sol-gel method in tailoring nanomaterial properties for specific biomedical applications, as inorganic-organic hybrids.

### Microwave-assisted method

Microwave-assisted method refers to a technique in which microwave irradiation is employed as a source of energy to facilitate or accelerate a chemical or physical process. In this method, the reaction mixture or precursor materials are exposed to microwave radiation, leading to selective and efficient heating. The use of microwaves can result in faster and more uniform heating compared to traditional methods, allowing for precise control over reaction conditions. This can lead to the rapid synthesis of nanomaterials with specific sizes, shapes, and properties [55]. Carregal-Romero et al. developed a cost-efficient synthetic microwave method to manufacture ultrasmall manganese ferrite nanoparticles with remarkable properties for multimodal imaging and therapeutic applications (Fig. 2d) [49]. The work represents a significant advancement in the synthesis of multifunctional nanoparticles with tailored properties for targeted biomedical applications.

### Green synthesis

Green synthesis refers to producing materials, chemicals, or nanoparticles by bacteria, fungi, algal species and certain plants. Jayandran et al. synthesized manganese nanoparticles using a green approach, where lemon extract served as the reducing agent and curcumin extracted from turmeric functioned as the stabilizing agent [50]. This green synthesis method emphasizes the use of naturally sourced components, aiming to minimize environmental impact and promote sustainability. Lemon extract and curcumin played roles as environmentally friendly reducing and stabilizing agents, providing a sustainable green pathway for the formation of manganese nanoparticles (Fig. 2e). Green chemistry methods have gained wide acceptance recently for synthesizing various metal nanoparticles owing to their eco-friendly nature and ease of synthesis. These methods have overcome the limitations associated with harmful chemicals and excessive energy consumption prevalent in industrial processes [71]. Biological agents such as bacteria, fungi, algae, and plants have been used for the green synthesis of manganese nanoparticles [72]. Green synthesis retains the advantages of nanomaterials and encompasses various beneficial properties, including antibacterial characteristics, natural reducing capabilities, and stability.

## Physicochemical characteristics

Manganese occurs in various oxidation states. For example, Mn^2+^, Mn^3+^, Mn^4+^, Mn^5+^, Mn^6+^, and Mn^7+^ can be tailored for different nanomaterials, each having different Mn valences and associated functions [56]. The most stable oxidation state is Mn^2+^ owing to its electron configuration of Mn^2+^ is [Ar] 3d^5^ [57]. When manganese forms Mn^2+^, it loses two electrons from the 4s orbital [58]. This results in a half-filled 3d orbital, which is more stable owing to the enhanced exchange energy and symmetry of the half-filled subshell. Additionally, manganese is transported across divalent metal transporter-1 (DMT1) in the body, primarily in the divalent form [59]. As a water-soluble ion, Mn^2+^ is rapidly excreted via the kidneys. In biological systems, Mn is an essential micronutrient for maintaining the normal functioning of cells and tissues and is crucial to brain growth and development and mostly use it as a mechanism to cope with oxidative stress [60]. Therefore, Mn nanomaterials hold promise in diverse biomedical applications, where synthesizing different shapes or valences of Mn-derived biomaterials can be applied to cancer diagnosis and therapeutic agents.

### Biological activities

Mn is an essential micronutrient for growth and the cellular regulation and can induce oxygen production from endogenous H_2_O_2_ in tumors, aiding in overcoming tumor hypoxia [61]. Moreover, MnO_2_ can be converted to Mn^2+^ through reduction by endogenous glutathione (GSH) resulting in the depletion of GSH in tumor cells and an increase in intracellular reactive oxygen species (ROS) levels, ultimately promoting tumor cell death [62]. Thus, Mn-derived biomaterials can regulate the TME via oxidation-reduction reactions. Mn^2+^ promotes dendritic cell maturation and activates the cGAS-STING pathway, initiating antitumor immune responses [63]. Excessive ROS production via redox cycling and the Mn^2+^-mediated Fenton reaction can generate highly toxic •OH, contributing to cancer treatments [64].

Mn is an activator of several enzymes, which can be separated into two categories: Mn metalloenzymes and Mn-dependent enzymes. Mn metalloenzymes include arginase, glutamine synthetase (GS), phosphoenolpyruvate decarboxylase, and Mn superoxide dismutase (Mn SOD). Mn-dependent enzymes include oxidoreductases, transferases, hydrolases, lyases, isomerases, and ligases [65]. Mn SOD is a key cellular antioxidant enzyme, primarily responsible for reducing ROS. It acts as an antagonist for the initiation and promotion of carcinogenesis [66]. The Mn-dependent enzyme G5 catalyzes the formation of glutamine from glutamate. Numerous cancer cells depend on glutamine, unless they express GS, making Alanine-serine-cysteine (ASC) amino acid transporter 2 (*ASCT2; SLC1A5*) a potential target for cancer chemotherapy [67]. Oxidative stress can be assessed by measuring the levels of antioxidants (glutathione or metallothionein), and the abundance of proteins (G5) which are extremely sensitive to oxidative stress [68]. Arginase is an Mn-dependent enzyme that catalyzes the hydrolysis of arginine to ornithine in the urea cycle. Arginase is important in normal and cancer cells [69]. Consequently, Mn participates in cell proliferation, stem cell maintenance, and inhibition of cancer cell migration.

Mn mediates cGAS-STING activation and is associated with ferroptosis [70], a type of non-apoptotic cell death dependent on iron and reactive oxygen. MnCl_2_ promotes mitochondrial ROS generation and lipid peroxidation, inducing ferroptosis by inhibiting dihydroorotate dehydrogenase (DHODH), an essential molecule in ferroptosis of tumor cells. This phenomenon can be explained by the production of type I interferons (IFNs) through Mn^2+^-mediated cGAS-STING signaling. IFN facilitates the function of DHODH and induces ferroptosis in tumor cells. However, inhibition of the cGAS-STING signaling pathway or administration of type I IFN can restore DHODH expression and prevent MnCl_2_-induced ferroptosis. These biological activities indicate that Mn and its isoforms have antineoplastic activities and the potential for developing novel therapeutics. Physiologically, Mn is involved in protein metabolism and regeneration of the connective tissue.

### Manganese in biomaterials

Various therapeutic techniques targeting tumors often rely on physical or chemical methods to excise tumors, leading to organ or tissue defects [71, 72]. Numerous biomaterials play pivotal roles in tumor treatment, organ tissue regeneration, and organ function reconstruction. Owing to its unique photothermal therapeutic capabilities and osteogenic differentiation potential, Mn plays a crucial role in extracellular matrix synthesis. Using advanced 3D printing technology to manufacture bioactive scaffolds exclusively doped with Mn ions, has shown considerable potential for bone tissue regeneration [73].

Mn-ion-doped bioactive glass-ceramic scaffolds (Mn-BGCs) exhibit marked bone-forming activity by stimulating osteogenic differentiation in vitro [74]. Additionally, they possess the capability to eliminate tumor cells via photothermal effects. This Mn-BGC approach concurrently addresses the challenges in photothermal tumor therapy and bone regeneration. The strategic integration of Mn ions with tissue engineering scaffolds presents a sensible approach to address bone tumors, aiming to enhance treatment outcomes and quality of life for patients, highlighting the excellent biocompatibility of Mn.

Wu et al. pioneered the incorporation of Mn-doped calcium silicate nanowires into sodium alginate hydrogels to create a dual-functional composite hydrogel for postoperative treatment of malignant melanoma [75]. This innovative platform has excellent biocompatibility, high photothermal conversion, and wound healing effects. It also serves as a multifunctional biomaterial with the ability to both eradicate tumors and facilitate skin tissue regeneration.

Owing to the prominent bioactivity of Mn, MnO_2_ nanoparticles (MnO_2_NPs) have demonstrated various biological functions that directly regulate tumor growth. To date, efforts have been dedicated to manufacturing various Mn oxides, sulfides, and hybrid nanostructures with potential applications in biological imaging and cancer treatment. The versatility of the MnO_2_NPs showcases their potential role in reshaping the landscape of cancer therapeutics and diagnostic imaging.

In conclusion, the integration of Mn ions into biomaterials has emerged as a revolutionary strategy, offering a multifaceted approach for addressing both tumor therapy and tissue regeneration challenges. This research frontier expands our understanding of the diverse applications of Mn and holds promise for future advancements in the field of medical biomaterials.

## Toxicity of manganese derived biomaterials

The toxicity of manganese-derived biomaterials, particularly inorganic nanomedicines, is influenced by several factors. These include the specific form of manganese, its concentration, and the interactions of the biomaterial with biological systems. Manganese is an essential element for biological functions, but excessive exposure or certain forms of manganese may pose risks. According to the National Academy of Sciences, the daily adequate intake of manganese for adult is approximately 1.8 to 2.3 mg. For children and adolescents, the recommended amount varies by age and gender, generally ranging from 1.2 to 2.2 mg [76]. Mn in excess causes a variety of neurotoxic effects, but typically does not present a risk to healthy adults if the daily intake from the diet remains below 9.2 mg. However, The World Health Organization has not calculated provisional maximum tolerable daily intakes for manganese [33]. It is noteworthy that in studies involving manganese derived nanoparticles used in MRI, researchers found that higher doses of MnO (785 µg Mn/ml) exhibited strong toxicity in mice [77]. Moreover, a study on rats showed that the optimal dose of MnCl_2_ for manganese-enhanced MRI is between 150 and 300 nmol, beyond which the retinal ganglion cells are likely to die [78]. Furthermore, the toxicity of manganese-derived biomaterials is not only dose-dependent but also significantly influenced by the valence states of the manganese ions. For instance, MnO_2_, containing Mn^4+^ ions, showed higher efficacy in killing cancer cells compared to Mn^2+^ ions at the same manganese content [79]. This enhanced cytotoxicity is attributed to GSH depletion by MnO_2_. In summary, different valences can exhibit discrepant toxicity profiles.

In the context of inorganic nanomedicines, which often involve nanoparticles or nanomaterials, their toxicity can be influenced by their physicochemical properties, such as size, shape, surface charge, and coating materials. These properties play a crucial role in interactions with cells and tissues. Some studies have demonstrated that Manganese Oxide Nanoparticles (MON) can serve as a CDT agent, exhibiting cytotoxicity towards tumor cells through a H_2_O_2_/GSH-dependent Fenton-like reaction at certain doses. Fortunately, the impact on normal cells is negligible, as the lower levels of H_2_O_2_/GSH in normal cells, compared to tumor cells, result in lower cytotoxicity of MON towards normal cells [80].

To assess toxicity, researchers conduct comprehensive studies, including in vitro and in vivo experiments, to understand the impact of manganese-derived nanomedicines on cellular functions, tissue responses, and systemic effects. Evaluations consider factors like biodistribution, biocompatibility, and potential adverse effects. The renal and hepatic pathways serve as common routes for nanoparticle excretion, contributing to the reduction of toxicity. In studies examining the biological effects of nanomaterials, particular attention has been directed toward evaluating liver and kidney functions. Feng et al. reported that, upon intravenous administration of Fe_5_C_2_@MnO_2_ nanocatalyst to mice, a time-dependent clearance effect was observed, primarily mediated by the liver and kidneys [27]. Additionally, serum biochemical analysis and histopathological results of the heart, liver, and kidneys indicated no significant abnormalities in major organs compared to normal mice, highlighting good tissue compatibility of the samples. Nanomaterials have become primary candidates for various biomedical applications, and manganese dioxide (MnO_2_) is particularly favored for its low toxicity. However, the long-term toxicity of these materials remains unknown and requires further research. Therefore, assessing the toxicity of different nanomaterials is crucial for understanding their potential clinical significance.

## Manganese-derived biomaterials for tumor diagnostic applications

Manganese-derived biomaterials have been reported to be used for various tumor diagnostic applications such as MRI, PT, PA, US imaging, multimodal imaging, fluorescence quenchers, and biosensors, all of which are discussed in the following subsections.

### MRI

Since MRI was first proposed by Lauterbur in 1973, it has become an invaluable tool for the detection, monitoring, and early diagnosis of various tumors [16]. MRI relies on the absorption and emission of radiofrequency energy by atomic nuclei in the presence of an external magnetic field. Hydrogen atoms are often used to enhance MRI signals, and the antenna receives signals from the tissues. Despite MRI offering high spatial resolution, its low sensitivity remains a significant limitation. Traditional contrast agents like GBCAs, while enhancing MRI visibility, often cause adverse health effects and are not suitable for all patients owing to their potential toxicity [8, 81, 82]. In contrast, manganese ions (Mn^2+^), with their paramagnetic properties, offer a safer alternative. They can effectively shorten the T1 relaxation times of water protons, enhancing signal intensity on T1-weighted MRI images without the health risks associated with GBCAs [83]. Mn^2+^ also impacts T2 relaxation times to a lesser extent, supporting versatile imaging applications [76]. Therefore, Mn nanostructures have been used as alternative CAs in T1-weighted MRI to obtain brighter images, enhancing diagnostic accuracy while avoiding adverse effects. The biocompatibility and strong paramagnetic properties of Mn^2+^ allow for short circulation times, preventing Mn atoms inside the nanoparticles from contacting the external water environment and minimizing proton relaxation. Consequently, Mn-based nanomaterials offer promising alternatives for MRI applications owing to their unique properties, such as Mn-DPDP (Teslascan™), employed in clinical settings to enhance liver and pancreatic imaging [84].

**Fig. 3:**
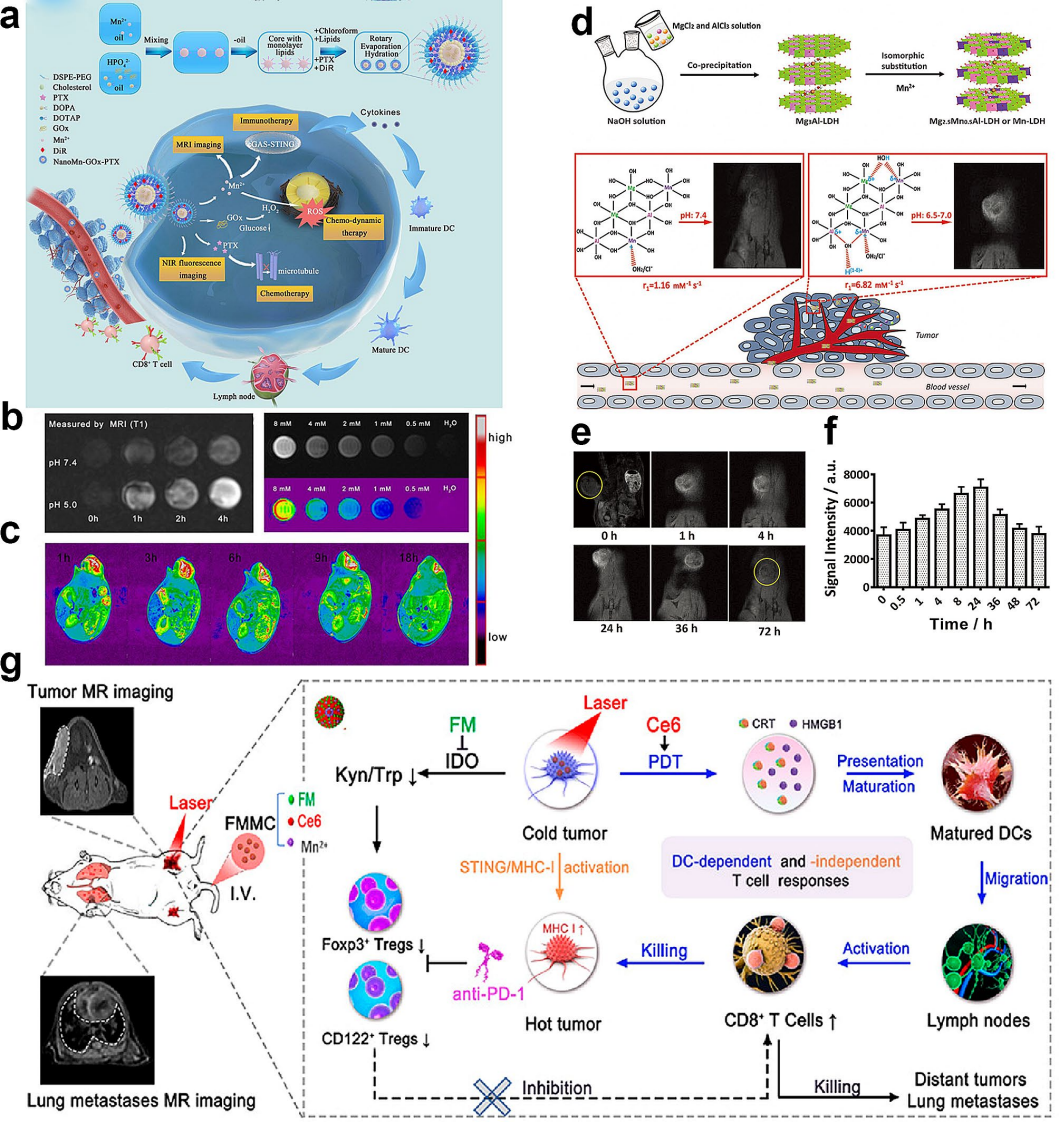
Manganese-derived biomaterials for enhancing magnetic resonance imaging (MRI) diagnostics in tumors. (**a**) Schematic illustration of the synthesis of NanoMn-GOx-PTX and its application for MRI contrast. (**b**) T1-weighted MRI of NanoMn-GOx-PTX for time gradients and concentration gradients in vitro (**c**) T1-weighted MRI images of mice injected with NanoMn-GOx-PTX in vivo [89]. . Copyright 2022, ELSEVIER. (**d**) Schematic illustration of synthetic procedure and its structure related multifunctional properties of Mn-LDH nanoparticles. (**e**) The contrast enhancement in T1-weighted MRI of the melanoma tumor of BSA/Mn-LDH nanoparticles in vivo. (**f**) The corresponding signal intensity variation is observed after intravenous injection of BSA/Mn-LDH nanomaterial over 72 h in vivo [90]. Copyright 2017, Willey. (**g**) Schematic illustration of FMMC for MRI-guided cancer therapy. Reprinted (adapted) with permission from [91] Copyright 2023 American Chemical Society

Recently, several Mn-derived biomaterials have been proposed as responsive CAs for MRI, including Mn oxides, Mn silicates, and Mn phosphates. Among these materials, MnO_2_ has garnered considerable attention owing to its established propensity for unstable transformations under biologically relevant pH conditions and redox-modulated states [85–87]. Nonetheless, a contentious discourse surrounds the behavior of alternative manganese oxide species employed in the context of MRI, notably MnO and Mn_3_O_4_. Although most researchers classify these two oxides as traditional non-responsive agents, a minority of publications posit their capacity for pH and/or redox responsiveness [88]. In 2022, Zhu et al. synthesized nanoscale precipitates containing Mn^2+^ and glucose oxidase (GOx) using the precipitation method. These were further combined with lipid membranes to construct a drug carrier, forming a pH-sensitive nanoplatform that delays the overload of manganese ions, enhancing the T1 contrast in MRI (Fig. 3a). In vitro experiments demonstrated that this nanoplatform tends to dissolve and release more manganese ions in acidic environments, enhancing the T1 contrast (Fig. 3b). In vivo experiments revealed that mice injected with the Mn^2+^ based nanoplatform exhibited brighter T1-weighted MRI images over time and concentration gradients (Fig. 3c). This nanoplatform can serve as a versatile tool for delivering proteins and anticancer drugs into cells, and the pH-responsive manganese nanoparticles can enhance tumor imaging sensitivity faster and more significantly than the clinically used GBCAs [89].

Notably, a subset of spherical MnO_x_ nanoparticles distinguished by their porous nanoshell architecture holds exceptional promise. This unique hollow nanostructure serves as an intrinsic reservoir, enabling simultaneous drug delivery and therapeutic-diagnostic integration. A recent breakthrough has been achieved by researchers from the University of Queensland and the University of New South Wales. Li et al. developed a strategy for the synthesis of manganese-based layered double hydroxide (Mn-LDH) nanoparticles that exhibited marked responsiveness to the TME characterized by a mildly acidic pH range of 6.5–7.0 (Fig. 3d) [90]. This heightened responsiveness makes Mn-LDH nanoparticles high-sensitivity CAs for tumor imaging, markedly enhancing the imaging capabilities of nanoscale MRI CAs (Fig. 3e). This was achieved by integrating Mn ions into degradable two-dimensional LDH nanoparticles, yielding Mn-LDH nanoparticles with an average diameter of approximately 50 nm. The Mn-LDH nanoparticles were precoated with bovine serum albumin (BSA) to prevent nanoparticle aggregation in physiological environments, such as aqueous solutions, cell culture media, and serum. Capitalizing on their small dimensions, these nanoparticles demonstrated augmented accumulation at tumor sites via enhanced permeability and retention effects (Fig. 3f), facilitated by the deformed vasculature of solid tumors. In the tumor-specific micro-acid environment, Mn ions on layered double hydroxide nanoparticles formed ‘transient’ manganese-coordinated water molecules (Mn+∙∙∙OH_2_). This induces a shortened distance between the paramagnetic center Mn ions and water molecules, consequently prolonging water molecule residence times. This phenomenon significantly amplifies the MRI contrast effect. Importantly, this innovative approach marks the pioneering development of a nuclear magnetic resonance imaging CA that exhibits high sensitivity to extremely weakly acidic conditions (pH = 6.5–7.0).

The notable paramagnetic properties inherent in Mn-derived biomaterials make them excellent candidates for enhanced MRI and establish their advantage in guiding tumor immunotherapy and PDT for cancer treatment [91]. The synergy between excellent anticancer efficacy and MRI capability makes Mn-derived biomaterials promising immune nanosculptors for cancer theranostics. In conclusion, the multifaceted attributes of these biomaterials, including their paramagnetism, MRI-guided therapeutic capabilities, and anticancer efficacy, highlight their potential as versatile tools for advancing cancer diagnosis and treatment strategies (Fig. 3g).

### Photothermal, photoacoustic, and ultrasonic imaging

Traditional diagnostic methods include various molecular imaging techniques such as ultrasonic, MRI, CT, and PETCT. However, tumors need to reach a certain anatomical size for CT visualization, which limits early detection of cancer and hinders continuous monitoring during treatment. Hence, there is growing interest in more advanced imaging techniques, such as PAI and PTI. While some traditional small molecule organic dyes, such as methylene blue (MB) and indocyanine green (ICG), possess effective near-infrared absorption properties and are used in organic optical materials, their inherent optical instability, high cost, and difficulty in modification substantially restrict their further development [92]. Manganese-derived biomaterials, owing to their exceptional light-absorbing and TME-responsive properties, can accumulate at tumor sites, and not only hold a prominent position in the field of MRI contrast CAs but also play roles in other imaging fields such as photothermal imaging (PTI), photoacoustic imaging (PAI), and ultrasonic imaging (USI).

**Fig. 4:**
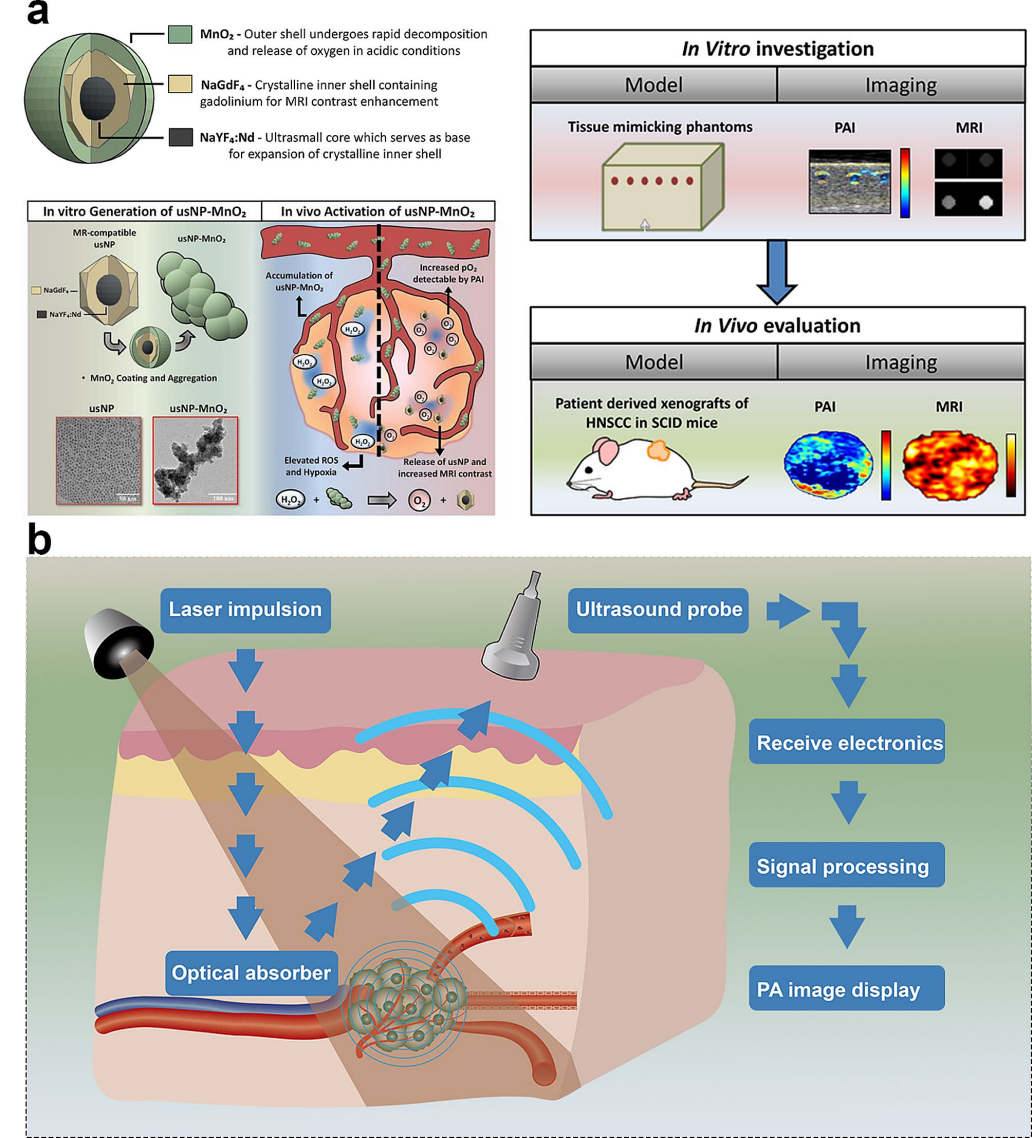
Manganese-derived biomaterials for photoacoustic, photothermal, and ultrasound imaging in tumors. (**a**) Photoacoustic imaging of hybrid manganese dioxide-coated ultra-small NaGdF4 nanoparticles for spatiotemporal modulation of hypoxia in head and neck cancer [47]. Copyright Rich LJ, Damasco JA, Bulmahn JC, Kutscher HL, Prasad PN, Seshadri M, 2020, MDPI, Fig. 1. Reproduced under CC BY 4.0 (https://creativecommons.org/licenses/by/4.0/). (**b**) The schematic illustrates the Photoacoustic Imaging (PAI) principle, where laser pulses are absorbed by tissues, generating acoustic waves that are detected to create detailed images with enhanced contrast and resolution

PAI, which converts absorbed light energy into acoustic waves, is a noninvasive medical imaging technique. PAI combines the advantages of high spatial resolution from ultrasound imaging and the ability to visualize structures with optical contrast based on tissue absorption properties [93]. MnO_2_ is a potential CA for PAI owing to its ability to enhance optical contrast, enabling precise tumor localization (Fig. 4a).

PTI depends on the conversion of absorbed light energy into heat using photothermal agents that selectively absorb specific wavelengths of light. This imaging technique highlights the distribution of photothermal agents in biological tissues. Mn^2+^ nanoparticles and other nanomaterials can serve as photothermal agents for cancer imaging as they can be engineered to target cancer cells. This enables the detection and visualization of malignant tissues with high specificity [47].

Ultrasound is a widely used, cost-effective, and nonionizing imaging modality. Ultrasound imaging relies on the generation of acoustic waves and their reflection from tissue boundaries with varying acoustic impedances [94]. The resulting images furnish morphological and flow information. When combined with PAI, ultrasonic imaging can offer comprehensive insights into tumor morphology and metabolism, facilitating a thorough evaluation of tumor characteristics (Fig. 4b).

Ren et al. developed a novel photothermal molecular imaging system and strategy for three-dimensional tumor-specific photoacoustic molecular imaging [95]. This system uses a manganese-centered texaphyrin derivative (manganese texaphyrin: MMn), known for its paramagnetism, strong porphyrin absorption, minimal loss of fluorescence emission, and protein-specific binding properties, which synergistically contribute to this imaging approach [96]. Unlike traditional porphyrins, MMn exhibits a long absorption wavelength, strong light absorption, and photobleaching resistance. At the core of this system, Mn (II) serves as a well-known fluorescence quencher and can enhance the near-infrared photoacoustic effect of MMn. Expanding on photoacoustic molecular imaging technology, researchers discovered that MMn yielded the highest PA signal intensity in RAW 264.7 cells, surpassing indocyanine green (ICG) in terms of photostability. Additionally, MMn enhanced photoacoustic imaging in a mouse model of prostate cancer, providing comprehensive 3D diagnostic information. Considering its potential for development into chemotherapeutic drug conjugates, MMn can be used as a relatively non-toxic standalone PA CA for diagnostic and therapeutic applications, or as a dual-mode (PA and MR) imaging agent for cancer diagnosis.

### Multimodal imaging

The ability to non-invasively visualize and assess tumors is crucial in modern oncology, however, each imaging modality offers unique advantages and inherent limitations. Multimodal imaging techniques that combine the strengths of different imaging modalities are gaining prominence owing to their potential to provide comprehensive information on tumor morphology and metabolism. In addition to MRI, PTI, PAI and USI, Mn-derived biomaterials have shown promising potential for the multimodal imaging of tumor cells [97]. The integration of multimodal imaging and synergistic treatment within a single platform has the potential to enhance both the therapeutic efficacy and diagnostic accuracy.

**Fig. 5:**
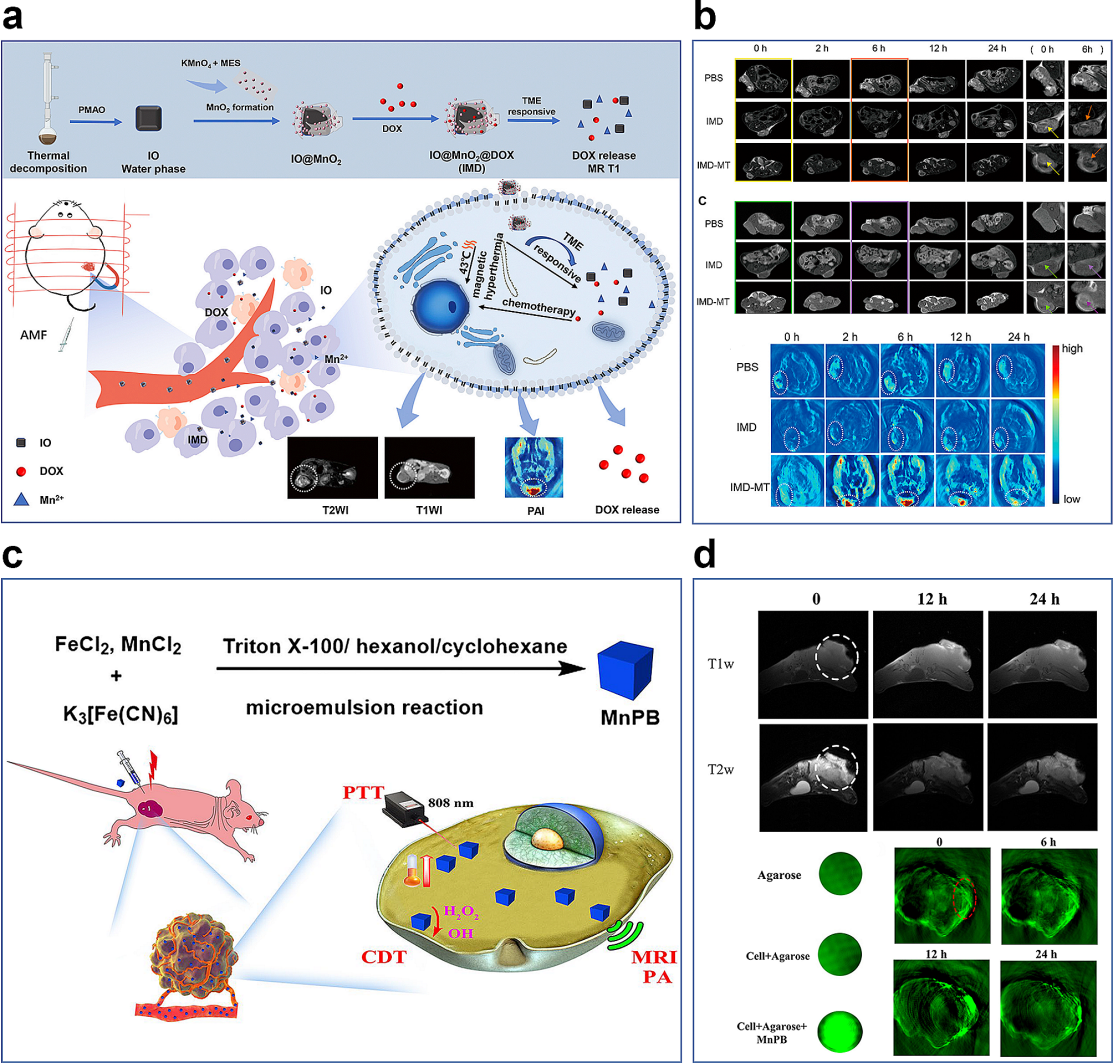
Illustration of the application of manganese-based materials for multimodal imaging in tumors. (**a**) Schematic illustration of the IMD for multimodel imaging guided-magnetic hyperthermia and chemotherapy [98]. Copyright Luo M, Lv Y, Luo X, Ren Q, Sun Z, Li T, Wang A, Liu Y, Yang C and Li X, 2022, Frontier, Fig. 1. (**b**) T2, T1 weight MR images, and PA imaging of IMD injection in vivo [98]. Copyright Luo, Lv, Luo, Ren, Sun, Li, Wang, Liu, Yang and Li., 2022, Frontier, Fig. 6. (**c**) The research scheme of MnPB NPs for T1/T2 weighted MR imaging, PA imaging and CDT/PTT co-therapy in tumor [99]. Copyright Tao Q, He G, Ye S, Zhang D, Zhang Z, Qi L, Liu R, 2022, BMC, Scheme 1 (**d**) T1, T2 MR, and Photoascoutic imaging of MnPB NPs in vivo [99]. Copyright Tao Q, He G, Ye S, Zhang D, Zhang Z, Qi L, Liu R, 2022, BMC, Figs. 3 and 4

To overcome the limitations of single modality imaging, Luo et al. successfully constructed a MnO_2_-based nanoplatform, demonstrating the versatility of Mn-derived biomaterials for multimodal tumor imaging and therapy [98]. The smart nanoparticles are composed of superparamagnetic IO, MnO_2_, and doxorubicin (DOX), and exhibit remarkable capabilities for high-efficiency T2-T1 MR imaging, switchable PAI, and TME-responsive DOX release (Fig. 5a). The IO@MnO_2_@DOX (IMD) exhibits promising potential as a convertible imaging agent for both MR and PA modalities. PA/MR bimodal imaging enhances the synergistic effects of magnetic hyperthermia therapy and chemotherapy. During preparation, water-soluble IO (Fe_3_O_4_) is synthesized via thermal decomposition and solvent exchange. Subsequently, MnO_2_ nanosheet-coated IO is obtained via the mild reduction of KMnO_4_ using 2-(N-morpholino) ethanesulfonic acid (MES). Finally, DOX is electrostatically adsorbed onto IO@MnO_2_ to form IMD. MnO_2_, a key component of IMD, serves as an efficient broad-spectrum fluorescence quencher, enhancing its applicability to fluorescence imaging (FLI), further expanding the diagnostic potential of this platform. MnO_2_ can respond to the TME by consuming excess GSH, and the release of Mn^2+^ enables the IMD to switch between different imaging modes. To assess the in vivo tumor diagnosis efficacy of the IMD, researchers conducted PAI experiments, confirming the accumulation and degradation of IMD in the tumor area. PAI outcomes were aligned with those obtained from MRI (Fig. 5b). This TME-responsive feature enhances the versatility of PA imaging, allowing for the dynamic monitoring of TME changes during treatment. The IO core of the IMD provides outstanding T2 MRI contrast. Simultaneously, released Mn^2+^ from MnO_2_ serves as a potent candidate for T1 MRI, further enriching the multimodal MRI capabilities of the platform. IMD encompasses imaging capabilities and integrates therapeutic functions that synergistically complement its imaging capabilities. High levels of GSH in the acidic TME promote IMD degradation, resulting in controlled DOX release at the tumor site. Moreover, magnetic hyperthermia enhances the anti-cancer activity of DOX in vitro and in vivo, effectively suppressing tumor growth and facilitating the delivery of nanomedicines to tumor sites.

In summary, the exploration of multimodal imaging using Mn-derived materials and imaging-guided therapy with Mn-derived nanocomposites represents a considerable advancement over Mn-based single-imaging and treatment methodologies. The Mn-doped Prussian blue nanoplatform, which incorporates multimodal imaging and chemodynamic/mild-temperature photothermal co-therapy, represents a significant advancement in tumor diagnosis and therapy (Fig. 5c) [99]. Although in vitro assessments of nanoprobes provide foundational understanding, extensive in vivo experimental validation is indispensable. These validations are crucial for substantiating the potential of nanoprobes in tumor treatment, paving the way for their clinical application (Fig. 5d). This comprehensive approach contributes to an enriched understanding of intricate tumor microenvironments and to the development of innovative and effective strategies for tumor diagnosis and treatment.

### Biodetection

Cancer cell detection is a challenging task. Sensitive biomarker detection in cancer cells enables early and rapid diagnosis and real-time monitoring of the progress of cancer treatment. Unfortunately, most current clinical techniques have low sensitivity and fail to detect early-stage cancer cells [100]. Consequently, biosensors have emerged as promising tools for the sensitive detection of cancer cells with a focus on cancer diagnosis. A prominent type of biosensor employed for cancer diagnosis relies on tumor markers, providing evidence of tumorigenesis and real-time information on tumor progression and metastasis. Biosensors can be classified into colorimetric, fluorescent, and electrochemical. Mn-derived biomaterials have been extensively explored for various biosensor applications. These materials, often derived from MnO_2_, offer unique properties that make them suitable for biosensing purposes [101]. Mn-based materials with enzyme-like activity capable of oxidizing chromogenic substrates are commonly used in the colorimetric detection, analysis, and processing of biomolecules [102, 103]. Owing to their strong optical absorption across the visible spectrum, MnO_2_ nanomaterials are frequently used as quenching agents in fluorescence biosensors [104].

#### Colorimetric biosensors

Colorimetric, electrochemical, and chemiluminescence methods can be used to detect components in biosensors. In contrast, colorimetric sensing methods can be used to detect analytes quickly and conveniently. Recently, nanostructured noble metals such as Au, Ag, and Pt, have shown potential for early cancer diagnosis via colorimetric biosensors. Colorimetric biosensors, which rely on color changes, detect the presence of cancer by observing color changes in the presence of a chromogenic substrate [105]. Although colorimetric assays allow visual detection without the need for expensive equipment, the high cost of noble metals and complexity of the assay process have limited practical applications. Different studies have indicated that the oxidative-like activity of Mn^2+^-derived biomaterials makes them suitable for developing easy, effective, and low-cost colorimetric detection assays for biomolecules [102, 106, 107].

**Fig. 6:**
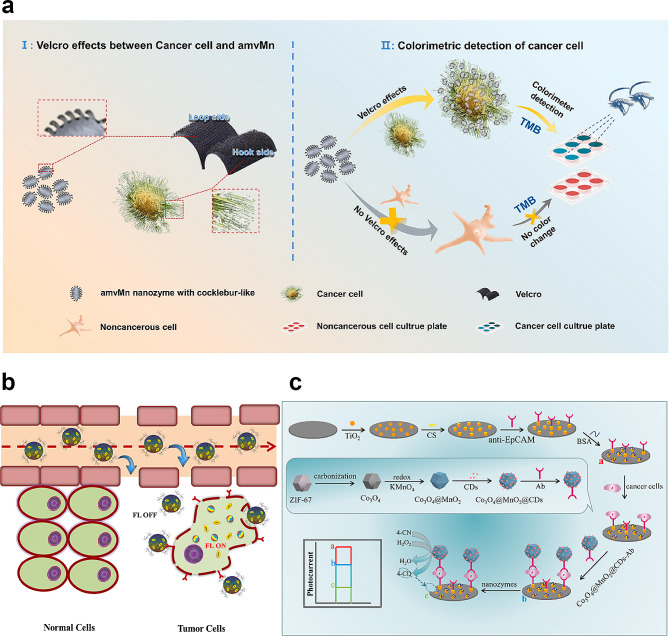
Visual representation of manganese-based biomaterials in biodetection for tumor-specific applications. (**a**) Schematic illustration of in vitro specific colorimetric detection of cancer cells by amvMn nanozyme with cocklebur-like morphology [111]. Copyright 2023, ELSEVIER. (**b**) Schematic illustration of MnO_2_-loaded fluorescent carbon nanogel remain non-fluorescent around normal cells but activate fluorescence in tumor cells [115]. Copyright 2019, ELSEVIER. (**c**) Schematic illustration of the PEC biosensor for the detection of cancer cells based on Co_3_O_4_@MnO_2_@CDs polyhedron with enhanced nanozymes activity [120]. Copyright 2023, ELSEVIER

Mn ions can oxidize colorimetric substrates such as TMB, OPD, and ABTS [108–110], resulting in a color change of the solutions. For example, Wang et al. introduced an innovative approach using an MnCO_3_-based precursor to create amorphous mixed-valence manganese (amvMn) nanozymes that underwent a simple calcination process to modulate the oxidation state and crystallinity of Mn within the nanozyme [111]. The amvMn nanozyme, characterized by its unique cocklebur-like morphology, specifically binds to cancer cells through a Velcro-like effect. Notably, amvMn nanozymes demonstrate oxidase-like properties, and experimental evidence suggests that their oxidase mechanism likely involves a mixed-valence state dominated by Mn (III), facilitating electron transfer between substrates and oxidants such as O_2_, effectively converting O_2_ into singlet oxygen (^1^O_2_). Moreover, they catalyzed the conversion of TMB to its blue form, enabling colorimetric detection of cancer cells. This groundbreaking work offers insights into optimizing nanozyme performance and inspires the development of equipment-free visual cancer cell detection methods (Fig. 6a).

Zhao et al. designed and presented a melanoma cancer diagnostic system based on MnO_2_-TMB [103]. They used the peroxidase-like activity of MnO_2_ to detect tyrosinase, enabling a simple, cost-effective, and rapid diagnosis of melanoma. Although some research on the role of Mn-based materials in colorimetric tumor diagnosis exists, further studies are needed to develop more cost-effective detection methods, making them more suitable for cancer screening and aiding in the early-stage detection of cancer.

#### Fluorescence biosensors

Nanomaterials offer significant potential for use in sensor applications, such as gold nanoparticles, carbon nanotubes and graphene quantum dots [112, 113]. However, these nanoparticles are often limited by low catalytic efficiency and the low concentration of H_2_O_2_ in the TME. Thus, manganese-based nanomaterials, due to their responsiveness to the TME, emerge as favorable choices for cancer diagnosis and therapy. In biosensors, MnO_2_ or Mn-based nanomaterials react with overexpressed intracellular GSH to generate abundant Mn^2+^ and release primers, providing a high-efficiency, wide-spectrum fluorescence quencher, consequently used to detect various analytes, including cellular GSH and microRNA [114]. Choi et al. demonstrated that MnO_2_ effectively achieves controlled drug release within fluorescent carbon nanogels (FCN) by quenching the fluorescence signal, and its superior response to GSH enables MnO_2_ to distinctly differentiate between normal and tumor cells, thereby exhibiting excellent tumor imaging capabilities (Fig. 6b) [115]. Chen et al. developed a multifunctional nano-platform (MnAs-ICG namospike) through electrostatic and coordination interactions [116]. This nanodrug self-assembles from ICG, manganese ions (Mn^2+^), and arsenate (AsO_4_^3−^). It effectively integrates MRI and FLI for synergistic photothermal/chemical/chemodynamic therapyArsenic trioxide (ATO) is an FDA-approved chemotherapeutic agent that faces challenges owing to its rapid renal clearance and severe systemic toxicity [117], necessitating combination therapy. Mn ions (Mn^2+^) are favored because of their inherent biocompatibility, high reactivity in Fenton-like reactions, and excellent MRI contrast ability. Mn^2+^ enhances biocompatibility, prolongs circulation, and targets tumor sites. Mn can also co-precipitate with arsenate (AsO_3_^3−^) to form manganese arsenide (MnAs) complexes via electrostatic interactions. MnAs remains stable at neutral pH and dissociates in acidic environments; thus, MnAs-ICG has good pH responsiveness, allowing controlled ATO release while improving drug loading efficiency. ICG is a near-infrared fluorescent dye used for FL and PTT. However, limitations in tumor diagnosis and treatment arise from its rapid elimination in vivo and unavoidable photobleaching. Mn^2+^ self-assembles with ICG via coordination interactions. The assembled MnAs-ICG responds to the TME and enables the on-demand release of Mn^2+^, AsO_4_^3−^, and ICG, effectively penetrating tumor tissues. ICG encapsulation via coordination interactions only prolong circulation time at tumor sites and enhance tumor-targeting capabilities compared to ICG alone. In conclusion, Mn^2+^ efficiently integrates a multifunctional therapeutic nanoplatform via interactions with ICG and ATO, serving as a bridging ligand.

#### Electrochemical biosensors

Fluorescent, colorimetric, and electrochemical biosensors are the three most commonly used types. Fluorescence and colorimetric diagnostic methods are time-consuming, expensive, have limited sensitivity, and rely on complex instruments. Therefore, the development of highly sensitive and cost-effective biosensors for improved diagnostic and therapeutic applications is critical for modern medicine. Electrochemical biosensors play an important role in the detection of tumor markers owing to their rapid response, miniaturization, and ease of operation [118]. In the pursuit of novel inorganic carriers for biosensor development and drug delivery, MnO_2_, especially owing to its high biocompatibility, tunable structure, electronic properties, and low cost attributed to the abundance of Mn on Earth, has garnered considerable attention.

Liu et al. designed a novel pH-responsive electrochemiluminescence immunosensor based on hollow MnO_2_ for the detection of avian leukosis virus subgroup J (ALV-J), associated with chicken tumors [119]. The hollow mesoporous nanosphere structure provides a higher surface area and lower density than bulk materials, leading to faster reaction rates. This innovation allows the new sensor to exhibit numerous potential advantages, including a low detection limit, high sensitivity, excellent reproducibility, and stability. HMnO_2_ nanospheres possess a pH-responsive delivery system that enables encapsulation, transportation, and release of target substances in weakly acidic environments, making them promising multifunctional delivery platforms. To create this immunosensor, reduced graphene oxide (rGO) functionalized with tannic acid (TA) was prepared and used as the sensor platform for loading the primary antibody (Ab_1_). Subsequently, the SiO_2_@MnO_2_ nanocomposites were used to prepare HMnO_2_, which was used to encapsulate Ru(bpy)_3_^2+^. Additionally, poly (diallyldimethylammonium chloride) (PDDA), poly (acrylic acid) (PAA), and a secondary antibody (Ab_2_) were used to enhance the detected ALV-J signal. This high sensitivity was achieved using GCE/rGO-TA/BSA/Ab_1_ as the sensor platform and Ab_2_-hMnO_2_-PDDA/PAA-Ru(bpy)_3_^2+^ as the label. Under optimal test conditions, the electrochemical chemiluminescence immunosensor exhibited a logarithmic current response to ALV-J concentrations ranging from 10^1.80^ to 10^4.30^ TCID_50_/mL, with a detection limit of 10^1.71^ TCID_50_/mL. Furthermore, this immunoassay demonstrated excellent analytical performance for quantifying ALV-J, with a low detection limit and high sensitivity, reproducibility, and stability, indicating its potential for clinical applications.

Among these, photoelectrochemical (PEC) biosensors are emerging and developing biomarker detection and analysis technologies for cancer diagnosis, which have the advantages of photochemical and electrochemical technologies. Liu et al. developed a novel Co_3_O_4_@MnO_2_ @CD polyhedron as a multifunctional nanozyme for PEC biosensing of cancer cells [120]. In the PEC biosensor, the Co_3_O_4_@MnO_2_ @CD polyhedron, acting as a nanozyme, quenches the photocurrent of TiO_2_ NPs and catalyzes precipitates, contributing to a dual-action mechanism for enhanced detection (Fig. 6c). These studies highlight the considerable efforts directed towards developing innovative and dependable methods for detecting tumors and their molecular products. Owing to its distinctive oxidative and catalytic activities, Mn has attracted the attention of numerous researchers, leading to the development of Mn-based materials for tumor diagnosis and treatment. However, it is crucial to acknowledge that to date, no confirmation of any MnO_2_ Nes based biosensor has been successfully employed in clinical settings. Consequently, in-depth in vivo studies are imperative to validate the safety and efficacy of MnO_2_ NE-based biosensor systems for application in human tumor diagnostics. This crucial step will provide essential insights into the future use of Mn-based materials in clinical practice.

## Manganese-derived biomaterials for tumor treatment applications

Conventional cancer treatment strategies involving Mn-based biomaterials include drug delivery, hypoxia alleviation, enhanced MRI, PDT, RT, and starvation therapy. Notably, ROS can induce tumor necrosis or apoptosis, creating additional possibilities for the use of Mn-based nanozymes in cancer treatment.

### Drug delivery

There are three traditional methods for treating tumors: radiotherapy, chemotherapy, and surgery. Most chemotherapeutic drugs are characterized by limited bioavailability, poor drug diffusion, cytotoxicity, low tumor therapeutic specificity, multidrug resistance, and serious side effects. However, under certain circumstances, drug delivery vehicles can improve bioavailability to specific areas, prolong the therapeutic effect, and reduce side effects [121, 122]. Therefore, the targeted delivery of chemotherapeutic agents to deliver drugs at the site of action has become the focus of scientific research [123]. Various inorganic nanostructures such as gold nanoparticles [124], platinum nanoparticles, and silver nanoparticles [125] have shown great potential in nanomedicine. However, the heterogeneity of the vascular system, the diversity of tumor types, and the variability of the tumor microenvironment often result in poor targeting by conventional nanomaterials. Utilizing strategies that exploit the acidic and hypoxic conditions of the tumor environment can be an effective approach. Recently, manganese-based nanomaterials (Mn-based NPs), including nanosheets, hollow structures, nanocages, and nanobubbles, have served as effective reservoirs for drug delivery. Mn can be engineered to produce functional nanomaterials with different valences and functionalities. Mn-based NPs have emerged as promising alternatives owing to their lower toxicity compared to other inorganic nanostructures. They have proven to be efficient systems for drug delivery, owing to their unique physicochemical properties, including ordered mesostructure, uniform porosity, high loading efficacy, and good biocompatibility [126].

**Fig. 7:**
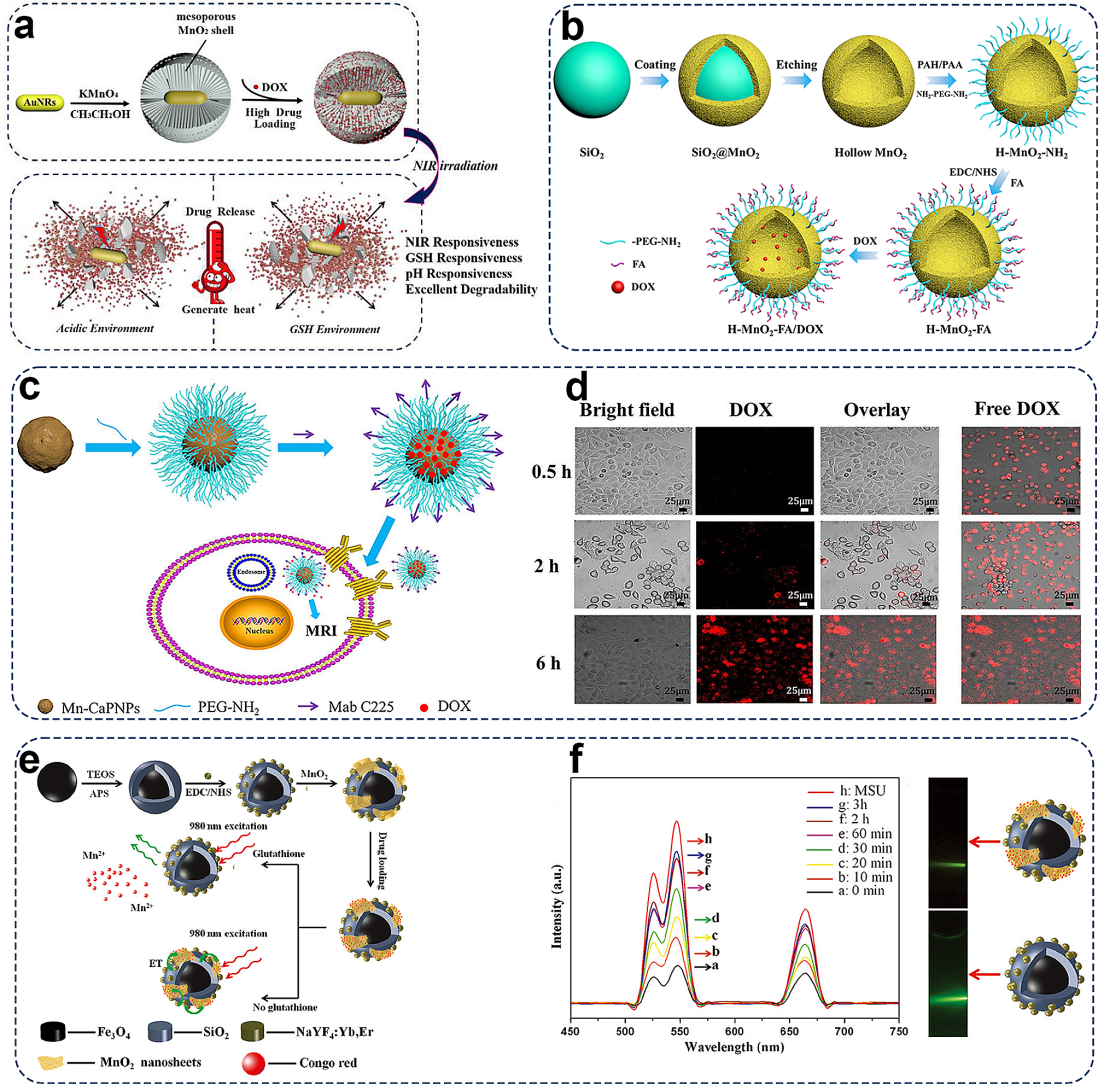
Visual representation of manganese-based materials employed in drug delivery systems for targeted tumor treatment. (**a**) Scheme of Preparation of Au/MnO_2_ Nanoparticles and the process of drug release. Reprinted (adapted) with permission from [127] Copyright 2019, American Chemical Society. (**b**) Schematic illustration for the preparation of hollow structured MnO_2_ modified with the targeted molecule for drug delivery. Reprinted (adapted) with permission from [128] Copyright 2020, American Chemical Society. (**c**) Schematic illustration of fabrication of Mn-doped calcium phosphate nanoparticle-based multifunctional nanocarrier. (**d**) Laser scanning confocal microscopy (LSCM) images of BxPC-3 cells incubated with Mn-CaPNPs-DOX and free DOX [129]. Copyright 2022, Springer. (**e**) Schematic illustration of the synthetic procedure for the preparation of the MSU/MnO_2_-CR drug delivery system. (**f**) PL emission spectra of MSU/MnO_2_-CR by adding 5 mM GSH at different release times [48]. Copyright 2014, The Royal Society of Chemistry

Zhang et al. designed a gold nanorod/mesoporous manganese dioxide (Au/MnO_2_) hybrid nanomaterial by combining gold nanorods (AuNRs) and MnO_2_ [127]. This hybrid nanomaterial serves as a multi-responsive (GSH-, pH-, and NIR responsive) platform for drug delivery. AuNRs have great potential as a nanoplatform for cancer therapy owing to their excellent photothermal properties. However, their rod-like structure leads to a low drug-loading capacity, and AuNRs are less stable. Under continuous NIR laser irradiation, they reshaped from rod-like to spherical structures. Under high-concentration GSH environments and acidic conditions, MnO_2_ can degrade and release loaded drugs. The combination of mesoporous MnO_2_ with AuNRs harnesses the photothermal effects of AuNRs and the GSH and pH responsiveness of MnO_2_ within a single nanoplatform. This results in a high drug-loading capacity and excellent GSH, pH, and NIR responsiveness. DOX is loaded onto Au/MnO_2_ hybrid nanoparticles via electrostatic interactions, hydrogen bonding, and physical adsorption, achieving considerably drug-loading efficiency (up to 99.1%). The in vitro drug release kinetics show sustained drug release in media with high GSH concentrations and acidic microenvironments, degrading mesoporous MnO_2_. The Au/MnO_2_ nanoparticles exhibit satisfactory drug release rates and excellent GSH, pH, and NIR responsiveness (Fig. 7a). The drug release behavior is markedly influenced by the exceptional photothermal effects and MnO_2_ degradation within the carrier. This platform holds promise for enhancing cancer treatment in photothermal therapy, drug delivery, and the TME.

With its hollow structure, H-MnO_2_ can be loaded with anti-tumor drugs, used for specific imaging and on-demand drug release in the TME, and regulate the hypoxic TME to enhance cancer treatment [128]. Cheng et al. designed and fabricated monodisperse H-MnO_2_ with a mesoporous shell for efficient targeted delivery of DOX, a well-known chemotherapeutic drug (Fig. 7b). To address the severe side effects associated with non-specific drug delivery in traditional chemotherapy, the integration of drug delivery with MRI has been proposed to achieve guided chemotherapy with efficient in vivo imaging. Zhou et al. devised a nano-platform for targeted drug delivery and cellular MRI [129]. The synthesized Mn-CaPNPs exhibited excellent performance in both Mn-based imaging and drug delivery (Fig. 7c). This innovative approach holds considerable promise for future therapeutic strategies by providing a dual-function platform for precise drug delivery and advanced imaging guidance (Fig. 7d).

In addition, Mn-based NPs can modulate the TME to enhance the chemotherapeutic efficiency. The TME is more acidic and contains a high concentration of GSH, which may severely influence the therapeutic effects of various anti-cancer treatments. Mn-based NPs can modulate GSH concentrations in tumor cells and introduce modifications to GSH metabolism, thereby ameliorating the hypoxic and antioxidant capabilities of tumors to enhance chemosensitivity. Furthermore, MnO_2_ nanosheets, a two-dimensional (2D) nanostructure of Mn-based nanobiomaterials with large surface-area-to-mass ratios, display unique and superior physicochemical characteristics compared to their bulk form [48, 130]. Zhao et al. developed an MnO_2_ nanocomposite by combining the magnetic and upconversion (UC) emission of Fe_3_O_4_@SiO_2_/NaYF4:Yb, Er hybrids modified with MnO_2_ nanosheets and showed a smart drug delivery system incorporating magnetic targeting, GSH-stimulated drug release, and UC luminescence tracking (Fig. 7e). Drug release can be monitored through UC luminescence, with luminescent intensity increasing over time as the release progresses (Fig. 7f). Unlike conventional drug delivery systems (DDSs), MnO_2_ NS-based nanoplatforms can function as controlled or on-demand DDSs and allow the tunable release of multiple encapsulated drugs in response to pH or different stimuli [131, 132].

### Alleviating intratumoral hypoxia

Radiotherapy and chemotherapy are currently the main treatments for most cancers. Radiotherapy generates radiation-induced inflammation and damages cellular proteins, lipids, and DNA. Radiation-induced oxidative stress activates ROS, whose primary targets are cellular mitochondria. Chemotherapy kills most cancer cells using chemotherapy agents and targeted therapy, and induces increased ROS levels via ROS-mediated apoptosis. Chemotherapy and radiotherapy share ROS as common downstream effectors. Elevated mitochondrial ROS levels promote oncogenesis, chemoresistance, radiation resistance, and metastasis, suggesting that the hypoxic regions in tumors can be resistant to radiotherapy and chemotherapy. Hypoxia is a hallmark of the TME. The TME is an integral part of cancer that fundamentally orchestrates tumorigenesis, disease progression, and treatment resistance.

**Fig. 8:**
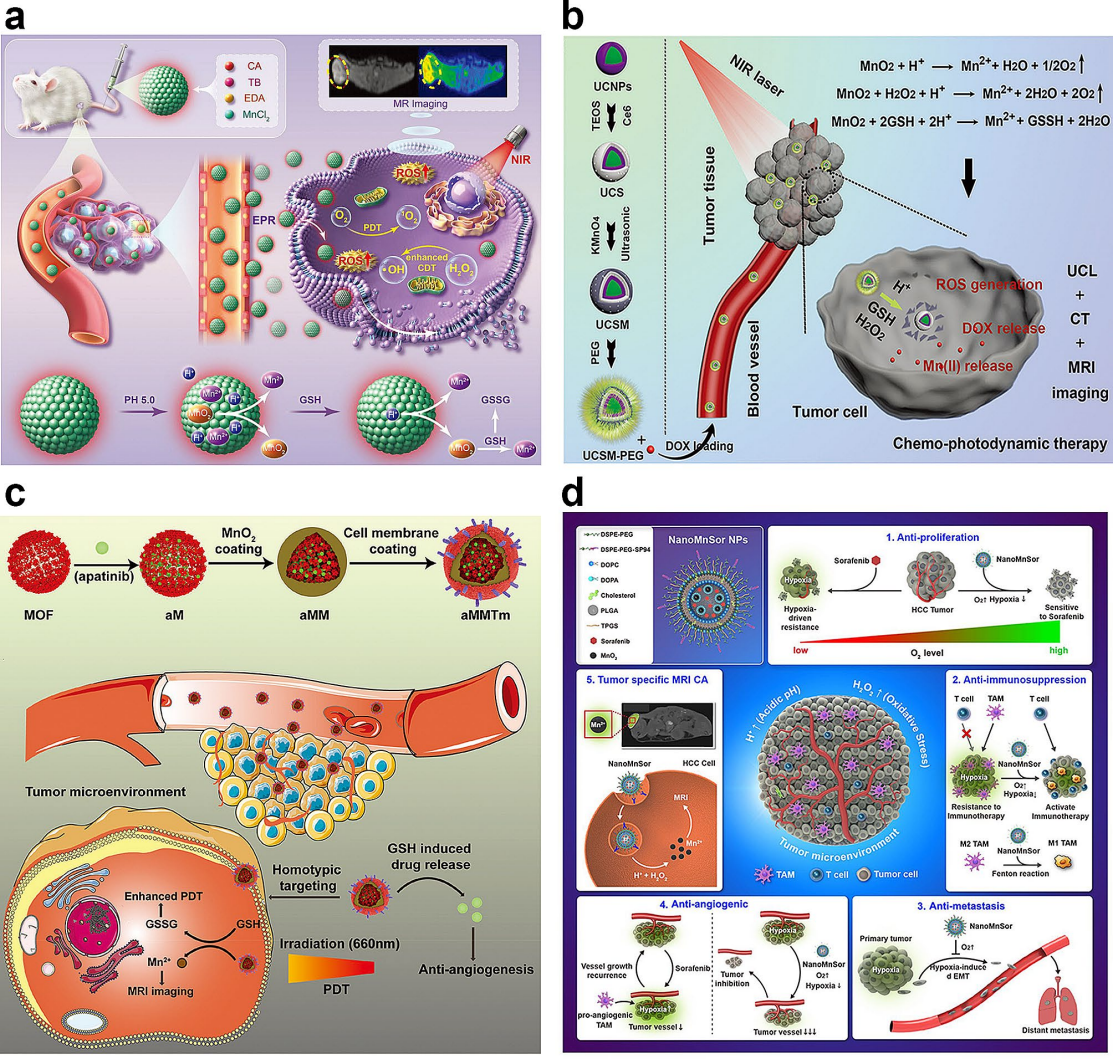
Illustration of the role of manganese-derived biomaterials in alleviating intratumoral hypoxia. (**a**) Schematic illustration of Manganese-doped TB CDs for MRI-guided tumor therapies response to TME [135]. Copyright 2023, Wiley. (**b**) Schematic illustration of UCSM-PEG for TME-enhanced chemo- photodynamic therapy and multiple bioimaging [141]. Copyright 2018, Wiley. (**c**) Schematic illustration of aMMTm preparation and proposed combination therapy of PDT and antiangiogenesis in TME [142]. Copyright 2019, Wiley. (**d**) Schematic diagram illustrating the regulatory role of manganese dioxide nanoparticles containing sorafenib (NanoMnSor) in the TME and its five associated functions. Reprinted (adapted) with permission from [143]. Copyright 2020 American Chemical Society

As Mn ions take multiple valence states from divalent to septivalent, Mn-derived biomaterials play a key role in the regulation of the TME via alterations in the redox status and ROS production [23]. The TME is characterized by oxygen-deficient solid tumors and a low pH environment (Fig. 8a). In the TME, cancer cells produce H_2_O_2_ and GSH in excess, which can significantly impair the immune escape of cancer cells and prevent tumor killing. Nanosized Mn oxides can react with H^+^, H_2_O_2_, and/or GSH to produce Mn^2+^, O_2_, and/or oxidized GSH to reverse the TME [133–135].

Recently, NP-based delivery systems such as inorganic NPs, liposomes, nanogels, lipid NPs, and polymeric NPs have been widely exploited for the treatment of various tumors [136–139]. Over the past decade, the utilization of Mn-based nanoplatforms, especially Mn oxide-based nanoplatforms, can improve the immunosuppressive TME, which has shown promising prospects in cancer therapy [133]. Compared with other metal nanoparticles such as Al, Zn, Ca, Mg, Fe, Au, Cu, and Co, Mn exhibits various antioxidant activities and offers noteworthy benefits in improving the PDT effect [140]. MnO_2_ converts H_2_O_2_ into water and oxygen, thereby alleviating hypoxia in the TME (Fig. 8b) [141]. Owing to the advantageous pH/H_2_O_2_/GSH-responsive behavior of MnO_2_, such nanocomposites can decrease the level of GSH and serve as highly efficient in situ oxygen generators to enhance PDT upon NIR irradiation [142]. Min et al. developed a multifunctional biomimetic metal-organic framework (MOF) nanoparticle via drug loading, MnO_2_ coating, and tumor cell membrane decoration for the anticancer combination therapy of PDT and antiangiogenesis (Fig. 8c).

To improve tumor-targeted treatment and address drug resistance, Chang et al. synthesized manganese dioxide nanoparticles containing sorafenib (nanoMnSor) as a nanoparticle drug carrier that can deliver MnO_2_ nanoparticles and sorafenib in the hepatocellular carcinoma TME to suppress and destroy hypoxia-driven tumors [143]. Sorafenib is a multi-kinase inhibitor that inhibits tumor growth and decreases tumor vascularization and metastasis, thereby improving overall survival. Furthermore, nanoMnSors reprogram the immunosuppressive TME by reducing hypoxia-induced tumor infiltration of tumor-associated macrophages. This promotes macrophage polarization toward the immunostimulatory M1 phenotype and increases the number of CD8^+^cytotoxic T cells in tumors, thereby augmenting the efficacy of the anti-PD-1 antibody and whole-cell cancer vaccine immunotherapies (Fig. 8d).

### Enhancing the efficacy of different therapies

#### Chemodynamic therapy

**Fig. 9:**
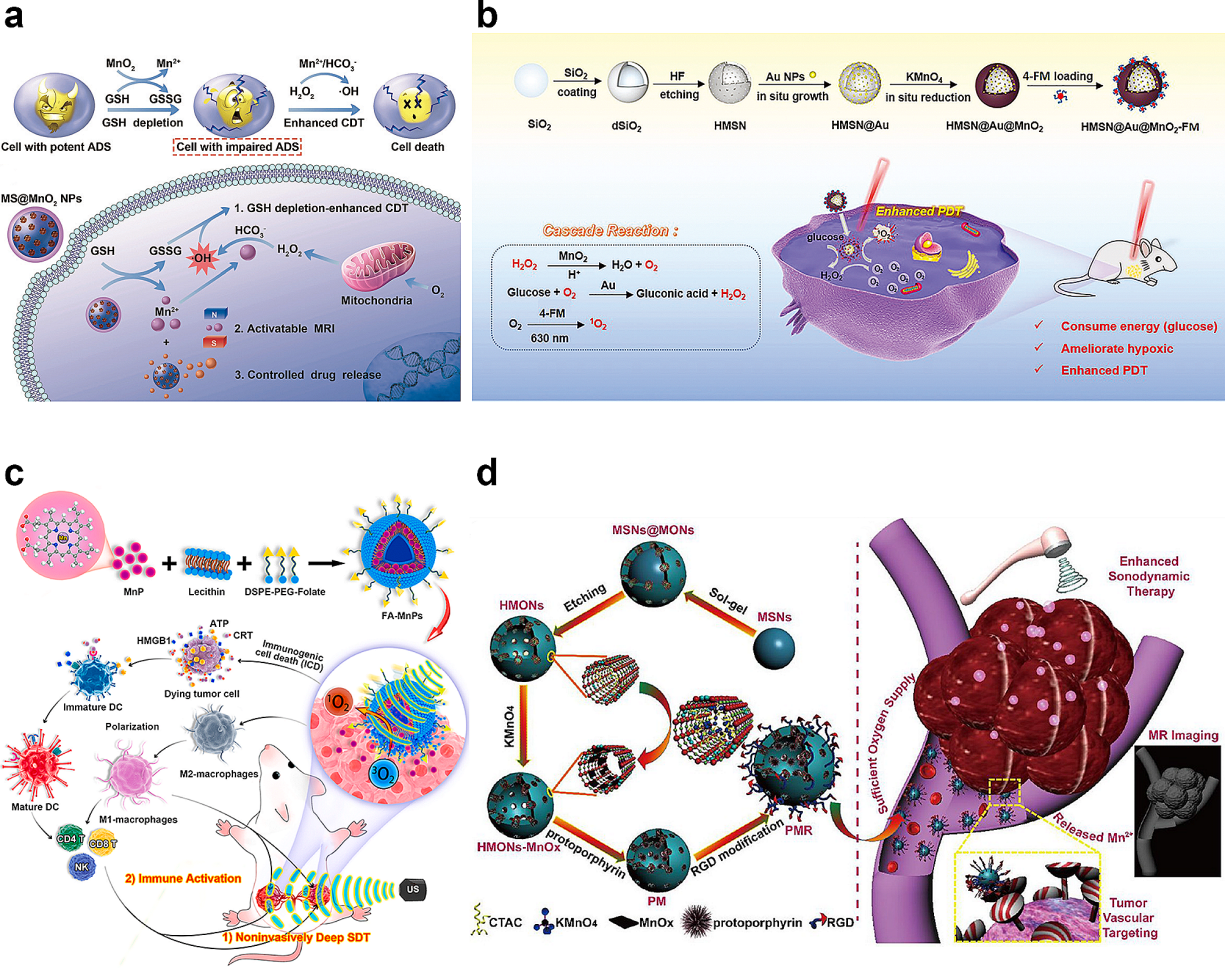
Manganese-derived biomaterials for enhancing the efficacy of various therapeutic approaches. (**a**) Schematic diagram illustrating the mechanism of MnO2 as a smart chemodynamic agent for enhanced CDT of cancer [26]. Copyright 2018, Wiley. (**b**) Schematic illustration of the synthesis process of HMSN@Au@MnO_2_-fluoresceinderivative nanoparticles and the mechanism of improving PDT [145]. Copyright 2021, Wiley. (**c**) Schematic depiction of FA-MnPs-mediated depth-penetrating SDT and immune activation for suppressing the tumors [154]. Copyright 2021, Wiley. (**d**) Schematic illustration of the construction of multifunctional nanoscale sonosensitizers and their MRI-guided and catalytic oxygen generation-enhanced SDT against cancer. Reprinted (adapted) with permission from [155] Copyright 2018, American Chemical Society

CDT, based on Fenton and Fenton-like reactions, generates the hydroxyl radical (OH•), a highly reactive ROS that has strong oxidizability to cells and target tissues [144]. Tumor cells in tumor tissue excessively produce H_2_O_2_, which leads higher H_2_O_2_ levels than in normal tissue. Thus, CDT relies on the higher expression of H_2_O_2_ in tumors. The H_2_O_2_ concentration in the TME is insufficient to continuously generate OH•. Therefore, increasing the level of H_2_O_2_ in the TME may enhance the efficacy of CDT. Mn^2+^ could mediate Fenton-like reaction with H_2_O_2_ and generated toxic OH• (Mn^2+^ + H_2_O_2_ → Mn^3+^ + OH• + OH-) for anti-tumor therapy, which damages tumor cellular macromolecules. In 2018, Yang and Chen presented a novel strategy to enhance CDT using MnO_2_ nanoparticles. MnO_2_ nanoparticles are synthesized in situ on thiol-functionalized mesoporous silica (MS) nanoparticles, resulting in MnO_2_-coated MS nanoparticles (MS@MnO_2_ NPs) suitable for chemotherapy and GSH-depletion-enhanced CDT (Fig. 9a) [26]. This study represents the first instance in which MnO_2_ has been identified as a CDT agent capable of inducing cancer cell death by generating cytotoxic OH•. Furthermore, MnO_2_ exhibits a higher capacity for generating OH• and demonstrates cytotoxicity, both in vitro and in vivo, compared to Mn^2+^-mediated CDT. This enhanced cytotoxicity is attributed to the depletion of GSH by MnO_2_. Subsequent research confirmed the efficacy of MnO_2_ in CDT.

#### Phototherapy

Phototherapies, including PDT and PTT, are light-mediated therapies for tumor cell killing. PDT relies on PS to transfer light energy to highly active singlet oxygen (^1^O_2_), which subsequently produces ROS and chemical energy to eliminate cancer cells. However, challenges such as hypoxic TME, low light-to-heat conversion efficiency, and the intrinsic toxicity of some photosensitizers limit their broader clinical application. Manganese-derived biomaterials, though not acting as traditional photosensitizers, significantly enhance these therapies by facilitating the controlled release of drugs and targeted delivery of photosensitizers, addressing the challenges of adverse tumor microenvironments and low photonic efficiency. Firstly, manganese materials can enhance the delivery of photosensitizers. For example, Yang et al. designed a HMnO_2_-PEG/C&D nanoplatform for precise drug loading and controlled release, demonstrating how manganese enhances phototherapy by improving drug delivery and efficacy [62]. Secondly, Mn ions can overcome the hypoxia of the TME via redox reactions, thus improving the efficacy of PDT and PTT and increasing their therapeutic efficacy in cancer treatment. The obtained H_2_O_2_ can generate O_2_ via CAT-like activity, enabling cascade catalysis and O_2_ cycling (Fig. 9b) [145]. Besides, Manganese significantly enhances the light absorption capabilities and photothermal conversion efficiency. Xu et al. found that compared to Pt and Pt@CeO_2_ nanostructures, Pt@CeO_2_@MnO_2_, due to their core–shell nanostructures, demonstrated high photothermal conversion efficiency (80%). After injecting Pt@CeO_2_@MnO_2_, the photoacoustic intensity in the tumor area remained at 70% of its peak value. The longer retention time of the tumor allows MnO_2_ nanomaterials to accumulate more within the tumor [146]. Zeng et al. developed a MnO_2_@Ce6@PDA-FA NPs encapsulating the photosensitizer Ce6. Under laser irradiation, MnO_2_ in combination with Ce6 releases a significant amount of oxygen, alleviating the hypoxic condition of the TME. Concurrently, the generated ^1^O_2_ is transformed into highly ROS, causing irreversible oxidative stress damage to tumor cells. Furthermore, combining manganese nanomaterials with suitable metallic components can achieve higher photothermal conversion efficiency. Xu et al. developed a manganese (IV) complex that shows about 71% efficiency in aqueous solutions under near-infrared irradiation [147]. Both in vitro and in vivo, the complex is highly biocompatible, easily cleared from the body, and holds significant potential for applications in photothermal therapy and MRI imaging.

To enhance the efficacy of PDT in the hypoxic TME, Zhang et al. designed and synthesized a novel Mn-carbon dot (Mn-CD) nanocomposite capable of in situ oxygen generation within tumors [148]. They developed a simple one-pot hydrothermal method to synthesize water-soluble Mn-CDs. The results demonstrate that the prepared Mn-CDs exhibit excellent near-infrared emission properties, stability, and efficient ^1^O_2_ generation. Furthermore, they possess acid- and highly H_2_O_2_-responsive characteristics, enabling the efficient catalysis of in situ O_2_ production in the TME, considerably improving the TME. Mn-CDs, with properties similar to those of MnO_2_, serve as nanosensitizers for in situ oxygen generation, and enhance the efficacy of PDT in hypoxic tumors.

Song et al. proposed a biocompatible and targetable PTT-PDT self-synergistic nanoplatform, RGD-BPNS@SMFN, for tumor eradication [149], by anchoring spherical manganese ferrite nanoparticles (SMFN) to black phosphorus nanosheets (BPNS), followed by arginine-glycine-aspartic acid (RGD) peptide modification. The RGD-BPNS@SMFN nanocomposite, based on temperature-dependent catalase-like behavior, includes manganese ferrite nanoparticles (MnFe_2_O_4_, MFN). These are biocompatible metal-based nanomaterials can be safely absorbed by the human body owing to the harmless breakdown of Mn^2+^ and Fe^3+^. They also possess potential photothermal and enzyme-like activities, enabling their effective participation in ROS catalytic cycles to combat tumor growth. BPNS are emerging nanocarriers for phototherapy. However, issues such as off-target effects, poor water solubility, rapid degradation, and fast clearance have limited the use of pure BPNS to achieve tumor ablation via PDT and PTT. Compared with pristine BPNS and SMFN, the nanocomposite material exhibits enhanced PDT and PTT performance, successfully eradicating HeLa cells. This synergistic effect is attributed to the self-promotion mechanism of the phototherapeutic process in the nanoplatform. This anticancer effect was further demonstrated in subcutaneous tumor-bearing nude mice.

#### Sonodynamic therapy

Sonodynamic therapy (SDT) similar to photodynamic therapy (PDT), stimulates sound sensitizers to generate ROS, inducing tumor cell apoptosis. SDT overcomes the limitation of penetration depth associated with PDT. However, SDT is also susceptible to the influence of the hypoxic TME, resulting in compromised therapeutic efficacy [150–152]. Therefore, SDT relies on the use of ultrasound in combination with sonosensitizers to induce cytotoxic effects in targeted cells. Numerous studies have substantiated the high sensitivity of Mn-based biomaterials to the TME and have proposed Mn-based nanomaterials as TME-activated sonosensitizers [153]. In addition, the Mn-based materials can specifically catalyze the overexpressed H_2_O_2_ in the TME to produce cytotoxic ·OH, owing to their high biological-like enzyme activity. Moreover, they can rapidly decompose in an acidic TME, and the released Mn ions offer strong MRI capabilities for imaging-guided SDT therapy. All of these factors collectively promote SDT and inhibit the development and progression of cancer.

Chen et al. devised a multifunctional nanosensitizer, FA-MnPs, which employs manganese-protoporphyrin (MnP) encapsulated within liposomes [154]. Liposomes serve as leading drug delivery platforms for cancer treatment by encapsulating hydrophobic agents in their bilayers. Folate (FA) receptors are overexpressed in several cancers, making them ideal candidates for enhanced drug delivery. Therefore, FA was integrated into the lipid bilayer of the liposomes, followed by the encapsulation of MnP to augment MnP accumulation and transfer within tumor cells (Fig. 9c). FA-MnP nanoparticles exhibit outstanding deep-tissue responsive SDT and concurrently activate SDT-mediated immune responses. Under ultrasound stimulation, FA-MnPs demonstrate high acoustic intensity, reaching depths of up to 8 cm in simulated tissues, while generating substantial amounts of singlet oxygen (^1^O_2_). Density functional theory calculations indicate that metal coordination within MnPs enhances US responsiveness. Effective deep-tissue-responsive SDT with FA-MnPs markedly inhibits the growth of superficial tumors in a triple-negative breast cancer mouse model and suppresses the growth of deep-seated lesions. FA-MnP-induced SDT further repolarizes immunosuppressive M2 macrophages into anti-tumor M1 macrophages and triggers immunogenic cell death (ICD) to activate dendritic cells, T lymphocytes, and natural killer cells, thereby initiating anti-tumor immunity and aiding the inhibition of tumor growth.

The oxidative stress induced by Mn-derived biomaterials increases ROS production. Based on the ability to facilitate SDT-induced ROS production, Mn-based biomaterials (MnO_2_, MnO, MnO_x_) can also be used to develop multifunctional nanosonosensitizers owing to their predominant properties [155]. In addition to enhancing SDT, the TME-responsive T1-weighted MRI capability provides the potential for therapeutic guidance and monitoring during SDT (Fig. 9d).

### Synergistic PTT/PDT/CDT

Monomodal cancer therapies are often unsatisfactory, leading to suboptimal treatment effects that result in either an inability to stop tumor growth and metastasis or prevent relapse. To ensure efficacy, nanoplatforms combining PTT and CDT should display low toxicity and good biocompatibility, be of an appropriate size for tumor accumulation, and exhibit excellent light absorption/light-to-heat conversion and catalytic potential.

**Fig. 10:**
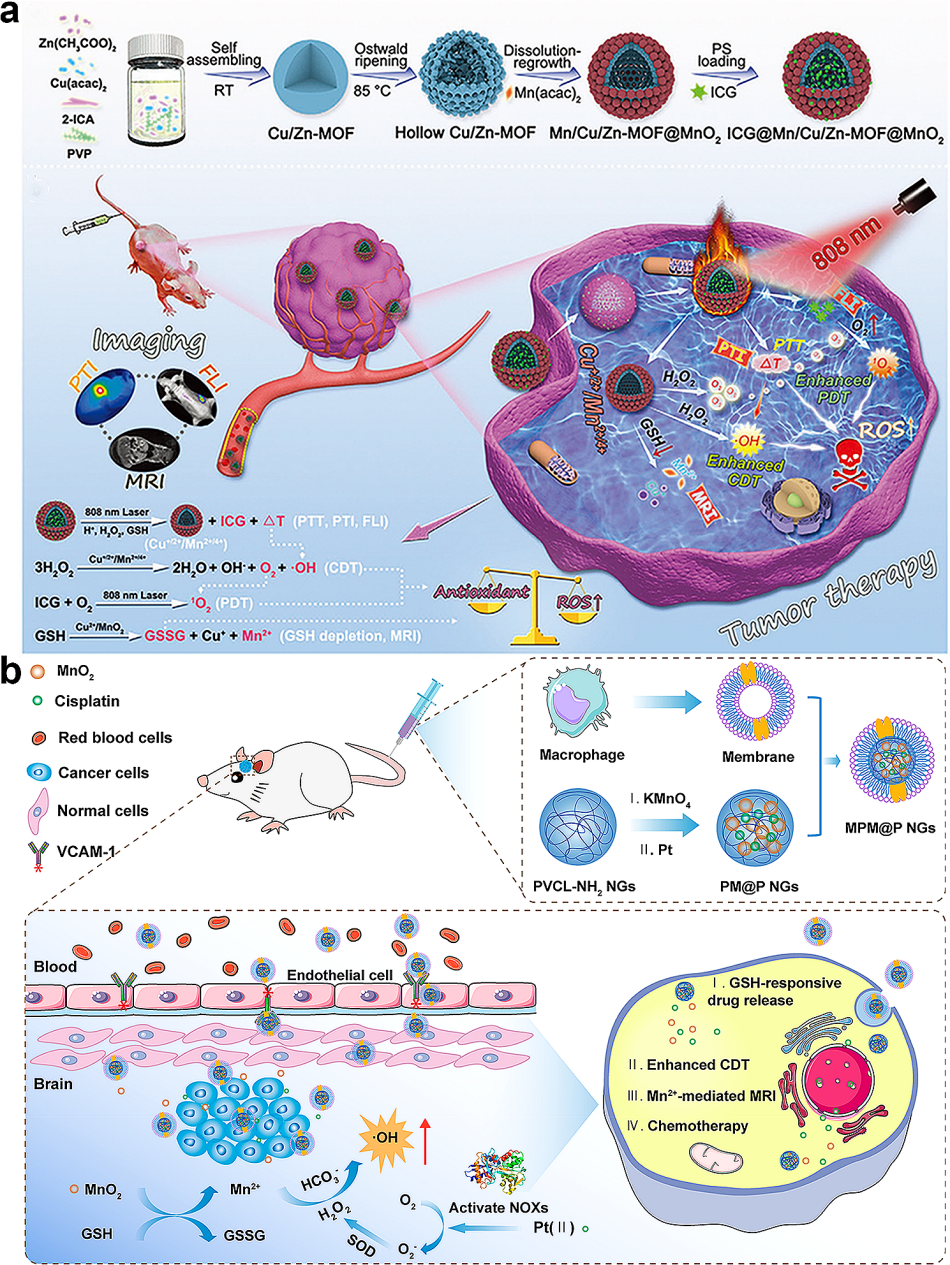
Manganese-derived biomaterials for the synergistic effects of photothermal therapy, photodynamic therapy, and chemical dynamic therapy. (**a**) Schematic illustration for the fabrication of ICG@Mn/Cu/Zn-MOF@MnO_2_ and the therapeutic mechanism for synergistic PTT/PDT/CDT [156]. Copyright 2021, Wiley. (**b**) Schematic illustration of the synthesis of Macrophage Membrane-Coated Pt/MnO_2_@PVCL NGs (MPM@P NGs) and its application for synergistic chemotherapy and CDT. Reprinted (adapted) with permission from [157] Copyright 2021, American Chemical Society

Cheng et al. introduced an innovative hollow nanoplatform (ICG@Mn/Cu/Zn-MOF@MnO_2_) for excellent trimodal imaging-guided synergistic PTT, PDT and CDT using a mixed-metal Cu/Zn-MOF as the precursor [156]. MOFs consist of metal ions and organic linkers. The nanoplatform synergistically enhances PTT/PDT/CDT, effectively inhibiting tumor growth under irradiation. Additionally, the release of substantial oxygen by MnO_2_ enhances ICG-induced PDT, while the generated Mn^2+^ produces cytotoxic •OH radicals that improve CDT, and also supports PTT (Fig. 10a). Notably, owing to Cu^2+^ and MnO_2_ depleted GSH and generated ROS, ROS-mediated CDT and other therapies were further enhanced. PTT-induced hyperthermia increased CDT efficiency, and TME activation enhanced MRI capabilities for further treatment guidance. This substantially enhances the therapeutic and diagnostic capabilities of Mn-based materials. For in vivo synergistic therapy of orthotopic glioma, Xiao et al. have developed macrophage membrane-camouflaged multifunctional polymer nanogels, co-loaded with MnO_2_ and cisplatin [157]. These nanogels are employed for combined chemotherapy and CDT with tumor-specific MR imaging. The dual pH- and redox-responsive release mechanisms enable targeted drug delivery, while the Mn^2+^ released from MnO_2_ depletes GSH in the TME, enhancing the efficacy of CDT and specifically targeting glioma cells (Fig. 10b). Zeng et al. have developed MnO_2_ nanosheets modified with polyethylene glycol-cyclic arginine-glycine-aspartic acid tripeptide (PEG-cRGD), which encapsulate chlorin e6 (Ce6) for synergistic PTT/PDT. The MnO_2_-PEG-cRGD nanosheets exhibit a high Ce6 loading capacity (351 mg/g), excellent photothermal conversion efficiency (37.2%), and outstanding colloidal stability [158]. Liu et al. developed a TME-responsive biodegradable nanoplatform using CaCO_3_/MnO_2_-based nanoparticles for synergistic PDT and PD-L1 immunotherapy [159]. Benefitting from the Mn^2+^ released during MnO_2_ degradation, which improves oxygen availability within the TME, the platform significantly enhances PDT while also potentiating immune response through PD-L1 blockade, illustrating the critical role of Manganese in synergistic cancer therapy.

### Tumor immunotherapy

Tumor immunotherapy, a treatment method that activates a patient’s immune response to spontaneously attack tumor cells, is considered the fourth pillar of modern cancer treatment [160]. Immunotherapy has advantages over traditional anti-tumor treatment methods in that it extends progression-free and overall survival [161]. Common clinical immunotherapies include adoptive T-cell therapy (ACT), chimeric antigen receptor T-cell therapy (CAR-T), immune checkpoint inhibitors (CPIs), and tumor vaccines. Among these, ACT is a promising treatment modality for leukemia [162], whereas CAR-T therapy is successful in treating B-cell malignancies [163]. Additionally, CPIs induce an anti-tumor immune response by counteracting suppressive immune checkpoint regulatory pathways [164]. Despite considerable progress in cancer immunotherapy, including the development of immune CPIs, such as those blocking the CTLA-4/B7 or PD-1/PD-L1 pathways, and their notable extension of the survival of patients with different cancers, major limitations persist for widespread clinical application of cancer immunotherapy. These limitations include low immunogenicity, limited specificity, low delivery efficiency, and potential off-target side effects. Therefore, to enhance the effectiveness of tumor immunotherapy, diverse strategies are necessary to stimulate or enhance the anti-tumor immune response in patients with tumors and alleviate immune suppression in the microenvironment [165, 166].

**Fig. 11:**
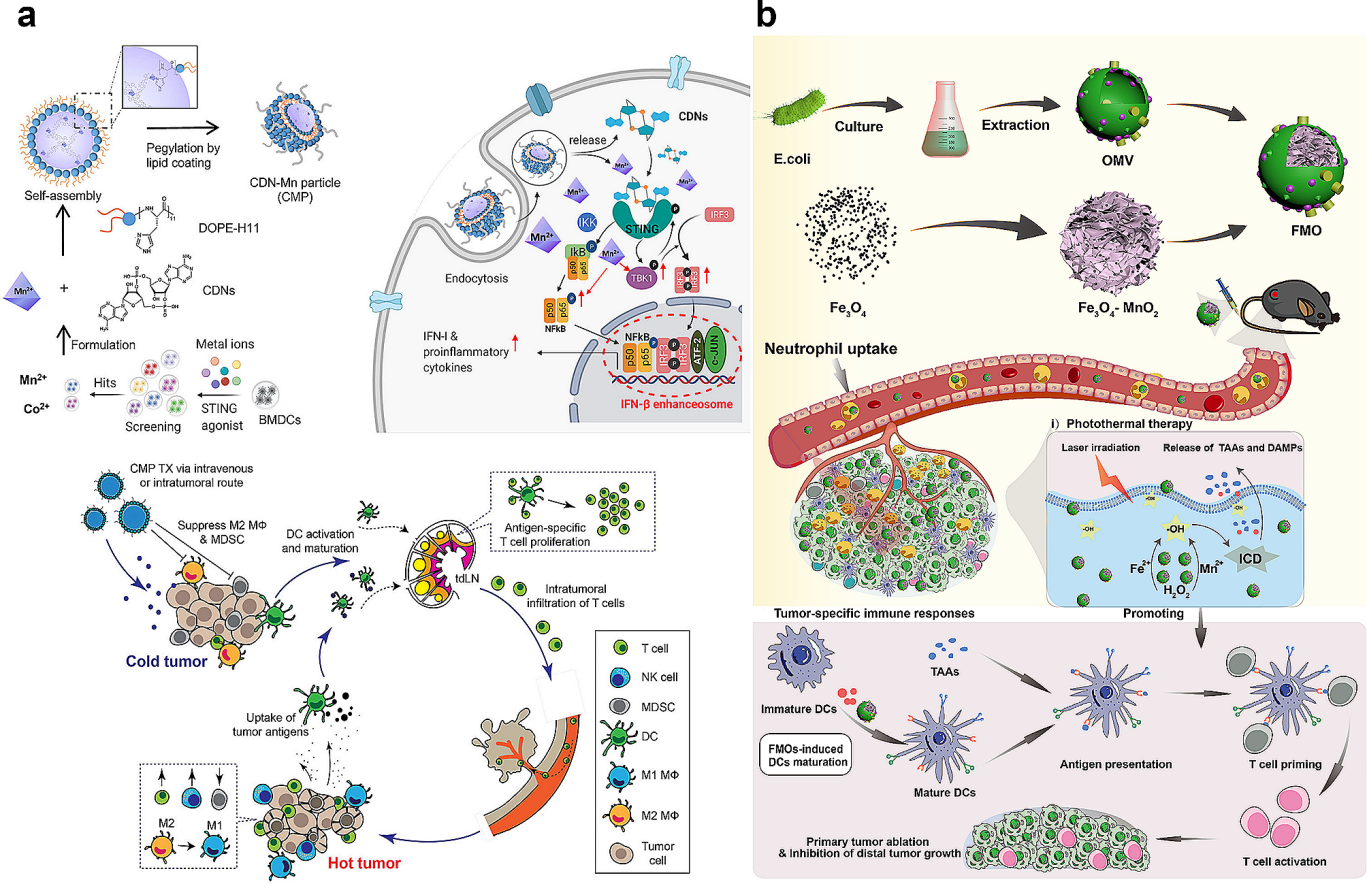
Manganese-derived biomaterials for enhanced cancer immunotherapy. (**a**) Amplifying STING activation with CDN-Manganese particles (CMP) for cancer metalloimmunotherapy. CMP, a complex structure of CDNs, Mn^2+^, and lipids, enhances STING activation, promoting anti-tumor efficacy by boosting immune responses and reversing the immunosuppressive tumor microenvironment [169]. Copyright 2021, Springer. (**b**) Schematic representation of OMV camouflaging Fe_3_O_4_-MnO_2_ NPs to generate FMO NPs and the mechanism of PTT-enhanced immunotherapy. Reprinted (adapted) with permission from [170] Copyright 2023, American Chemical Society

Manganese can be engineered into nanomaterials of various valence states, such as manganese oxide (MnO_x_) nanoparticles, which are extensively utilized as immunomodulators and immunostimulants in cancer immunotherapy [167]. In addition, manganese can also serve as a biocompatible nanocarrier for the targeted delivery of immunotherapeutic agents to immune cells, enabling dose reduction and enhancing immune response. Many research has demonstrated that Mn^2+^ is essential for activating cGAS-STING pathway by enhancing the sensitivity of enhancing the sensitivity of cGAS to detect DNA and promoting the synthesis of the second messenger cGAMP [63, 168]. This activation demonstrates its substantial potential for direct participation in tumor immunity. Sun et al. explored the application of manganese in immune processes, highlighting its role in enhancing the activity of STING agonists. Furthermore, it can serve as a multifunctional nanocarrier for tumor immunotherapy, targeting the delivery of immune activators to immune cells. (Fig. 11a) [169].

Liu et al. reported a multifunctional nanoparticle platform called Fe_3_O_4_-MnO_2_ (FMO) functionalized with drug-free bacteria-derived outer membrane vesicles (OMVs), which serves as an effective tool for cancer immunotherapy [170]. The synthesis process begins with the fabrication of Fe_3_O_4_ nanoparticles using a typical high-temperature thermal decomposition method. Subsequently, the reaction between potassium permanganate and oleic acid-coated iron oxide results in the formation of flower-shaped Fe_3_O_4_-MnO_2_ NPs. OMVs are collected from the culture supernatant of *Escherichia coli* and subsequently encapsulated onto Fe_3_O_4_-MnO_2_ NPs via ultrasonic treatment. *E*. *coli* targets deep-seated tumors using a “hitchhiking circulating neutrophils” strategy [171]. OMVs containing lipopolysaccharides are readily recognized and internalized by neutrophils. In this system, the modification of OMVs from *E*. *coli* enhances the accumulation of FMO NPs in tumor tissues via neutrophil-mediated targeted delivery. FMO NPs undergo reactions and decomposition at the tumor site, producing Mn and Fe ions, inducing ICD and O_2_ to overcome tumor hypoxia in the TME. Moreover, PTT induced by MnO_2_ and Fe_3_O_4_ indirectly stimulates systemic immunity by destroying tumor cells and promotes the enrichment of neutrophil NPs at the tumor site by enhancing the inflammatory response. In conclusion, the proposed multi-modal therapeutic system with targeted delivery capabilities achieves effective cancer immunotherapy to prevent tumor growth and recurrence (Fig. 11b).

### Imaging-guided therapy

Recent studies have underscored that current cancer treatments are often limited by severe complications arising from damage to normal tissues. For example, radiation therapy aimed at tumors often impacts a significant amount of surrounding healthy tissue, leading to radiation osteomyelitis [172]. High levels of antioxidants, such as GSH, in cancer cells limit the effectiveness of cancer treatments. Additionally, conventional materials used in biomedical applications, including various nanomaterials, frequently lack the necessary biocompatibility and responsiveness to the TME, which is crucial for effective therapy and imaging. Under such circumstances, Mn-derived biomaterials show a potent alternative owing to their excellent biocompatibility and unique chemical properties [173]. Various forms of Mn NPs, such as MnO, Mn_3_O_4_, and MnO_2_, serve as CAs in MRI, PAI, USI, dual-modal and multi-modal imaging, and imaging-guided tumor therapies. MnO_2_ materials exhibit reversible TME responsiveness and can be tuned to achieve different structures and morphologies, making them versatile for multiple applications such as biomedical imaging, drug carriers, biosensors, and cancer therapy [174]. MnO_2_ can react with H^+^, H_2_O_2_, and/or GSH to reverse the TME. Additionally, the Mn^2+^ produced during this process can be used as a T1 MRI contrast. Gao et al. demonstrated that a MnO_2_-entrapping dendrimer nanocarrier can co-deliver glucose oxidase and cyclic GMP-AMP for MRI-guided chemodynamic therapy, starvation, and immune therapies, thereby offering a multifaceted approach to tumor diagnosis and treatment [175].

**Fig. 12:**
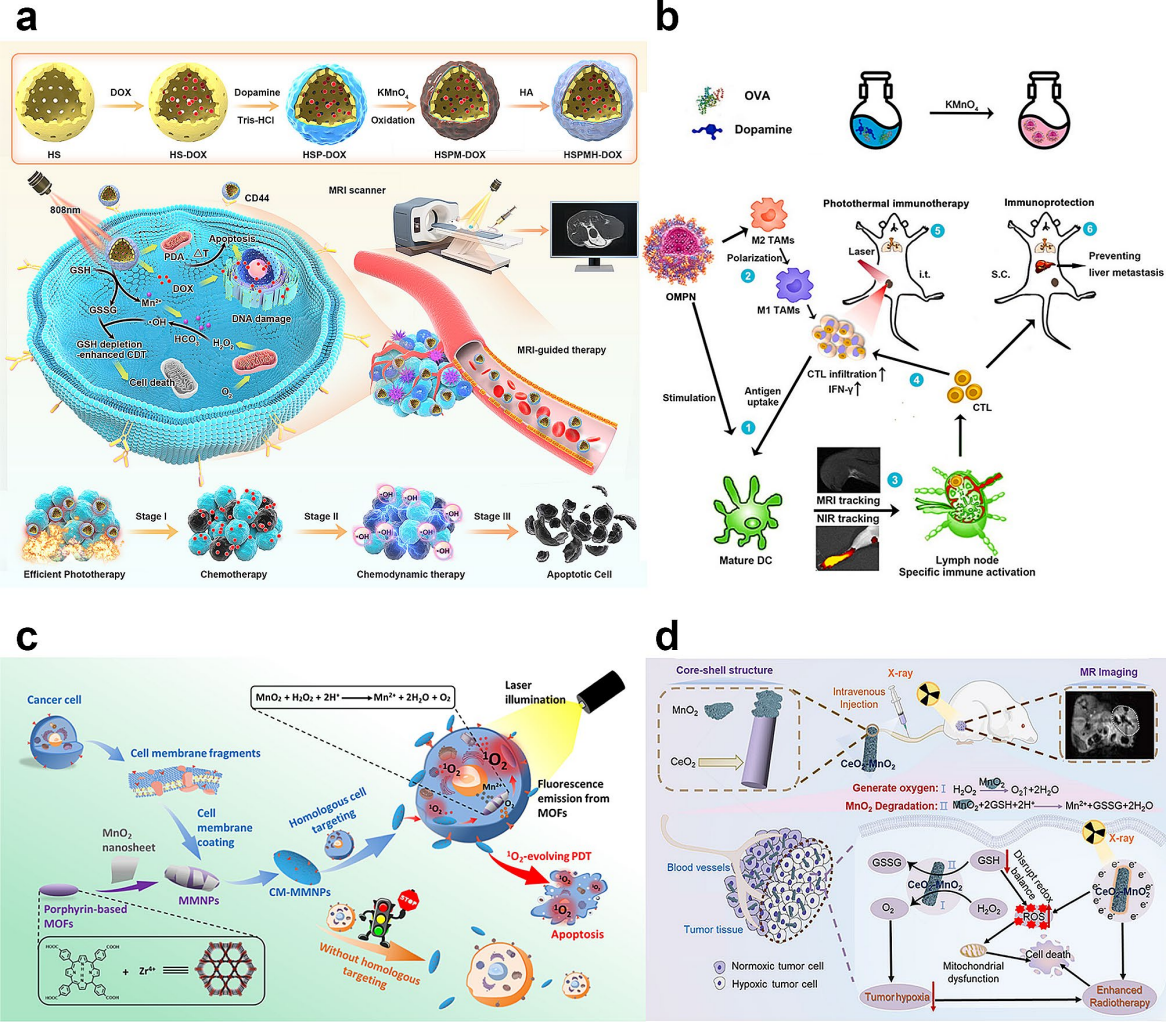
Application of manganese-derived biomaterials in guiding tumor therapy via magnetic resonance imaging. (**a**) Schematic illustration of synthesis process of HS@PDA/MnO2/HA-DOX (HSPMH-DOX) and TME-responsive activated magnetic resonance imaging and synergistic therapy [176]. Copyright 2022, Europe PMC. (**b**) Schematic illustration of the OMPN for cancer photothermal- immunotherapy enables real-time monitoring of DC migration via MRI [177]. Copyright 2021, Elsevier. (**c**) Schematic illustration of CM-MMNPs for MRI guide PDT. Reprinted (adapted) with permission from [178] Copyright 2019, American Chemical Society. (**d**) Schematic structure of CeO2-MnO2 and its synergistic mechanism for the treatment of hypoxic tumors [179]. Copyright 2023, BMC

Mn-based nanomaterials exhibit excellent MRI performance, making them suitable for applications such as tracking biological distribution, monitoring tumor treatment, and evaluating treatment outcomes. As shown in Fig. 12a, Shi et al. developed an intelligent dual-responsive nanocomposite (HSPMH-DOX) for MRI-guided synergistic chemo-PTT and CDT (Fig. 12a) [176]. The nanocomposite exhibits considerable therapeutic effects in PTT-, CDT-, and TME-responsive chemotherapy and enhanced synergistic effects in both in vitro and in vivo studies. Notably, MnO_2_ possesses nanozyme characteristics that enable MRI, controlled drug release, and enhanced CDT via GSH consumption. Furthermore, the prepared nanotherapeutics exhibit satisfactory biocompatibility and targeting capabilities, enabling precise tumor targeting via MRI-guided combined PTT, CDT, and chemotherapy. Xiao et al. developed a multifunctional nanovaccine (OMPN) containing ovalbumin, MnO_2_, and dopamine (OMPN) using a simple one-pot synthesis [177]. This nanovaccine eradicates primary melanomas and prevents liver metastases. Notably, this nanovaccine allows real-time monitoring of dendritic cell migration via MRI following vaccination, enabling the tracking of the immune process and assessment of the anti-tumor immune response (Fig. 12b). Thus, MRI-traceable therapeutic nanovaccines are promising candidates for cancer treatment. MRI-guided tumor therapy can address the shortcomings of the poor targeting ability of PDT. The introduction of Mn addresses the insufficient treatment efficiency caused by hypoxia in tumor cells and provides superior imaging technology. This enhances lesion contrast and tissue penetration, thereby improving the targeting ability of PDT treatment. Zhang et al. developed the MMNP nanostructure, which is an MnO_2_ nano-sheet-coated metal − organic framework core and cancer cell membrane shell [178]. The multifunctional nanostructure exhibits excellent dual-mode imaging and PDT efficiency, laying the foundation for the development of novel diagnostic and treatment platforms (Fig. 12c). Additionally, Mn-derived materials can enhance MRI-guided radiotherapy by alleviating the hypoxic TME. Pi et al. designed CeO_2_–MnO_2_ nanoparticles with a considerable radiosensitizing effect using a hydrothermal method [179]. CeO_2_–MnO_2_ exhibits anti-tumor properties and responds to GSH and H_2_O_2_, generating ROS and oxygen, enhancing X-ray absorption at the tumor site, and improving the cancer microenvironment (Fig. 12d). These nanoparticles also possess MRI functionality, enabling the precise localization of tumor lesions and enhancing anti-tumor effects. In conclusion, the diverse applications of Mn-derived biomaterials in tumor treatment, guided by MRI, demonstrate their pivotal role in addressing therapeutic challenges. These multifunctional platforms offer promising prospects for advancing precision medicine and improving cancer therapeutic outcomes.

### Applications of manganese-based materials in different tumor types

The efficacy of different therapeutic approaches differ among different tumor types. Conventional nanomaterials used in cancer treatment frequently encounter problems such as poor biocompatibility, inadequate targeting, and limited penetration into tumor tissues. In this context, manganese-based materials emerge as potent alternatives, offering significant improvements over these issues.

For PDT in lung cancer, Cao et al. developed an MnO_2_@Ce6-loaded mesenchymal stem cell nanoplatform [180]. These cells utilize MnO_2_ NPs to stabilize Ce6 in the bloodstream, modulate the TME, and upon laser irradiation, release substantial amounts of O_2_, alleviating TME hypoxia and promoting effective PDT. In addition, MnO_2_ can decompose into Mn^2+^, contributing to enhanced T1 contrast MRI by exhibiting high T1 relaxivity.

Most tumor immunotherapy exhibit poor performance against solid tumors owing to factors such as the immunosuppressive tumor microenvironment, low tumor immunogenicity, and inadequate T-cell infiltration. The efficacy of immunotherapy is largely constrained by the immunosuppressive TME. Mn-derived biomaterials, which serve as excellent endogenous O_2_ suppliers that react with H_2_O_2_/H^+^, demonstrate marked capabilities in modulating the immunosuppressive TME by overcoming tumor hypoxia [181]. For immunotherapy in bladder cancer, Wu et al. developed a novel MnO_2_@PDA NP loaded with ICG and α-PD-L1 [182]. This nanoplatform alleviates tumor hypoxia through catalytic conversion of MnO_2_ into Mn^2+^, and producing O_2_ within the TME and thereby enhancing the efficacy of immunotherapy. The generated oxygen not only supports adaptive immune responses but also improves the systemic delivery and efficacy of immune checkpoint inhibitors, offering a dual benefit of therapy enhancement and environment modulation.

Considering the diversity, heterogeneity, and recurrence of tumors, single-modality treatments often show limited efficacy, prompting investigations into the advantages of combined therapeutic strategies employing manganese-derived biomaterials. For comprehensive treatment in ovarian cancer, Mn-based materials are being explored for their potential to enhance conventional therapies. A Phase II clinical trial investigated the combination of MnSOD inhibitors with standard chemotherapeutic agents [183]. This synergistic approach significantly enhances the effectiveness of treatment by targeting MnSOD, a regulator crucial for the anchorage-independent survival and metastasis of cancer cells.

For head and neck cancer treatment, manganese-based materials have shown promising potential as radioprotective agents. A notable example of this application is the use of GC4419, a manganese superoxide dismutase mimetic, which has been evaluated in a phase IIb clinical trial [184]. The use of a manganese superoxide dismutase mimetic has shown promise in mitigating the duration, frequency, and intensity of severe oral mucositis in individuals undergoing radiation therapy for head and neck cancer. The findings revealed that GC4419 significantly enhanced all evaluated outcomes while maintaining an acceptable safety profile.

In light of the significant potential of Mn-based biomaterials in oncology, it is crucial to examine their current status in clinical trials to understand their practical applications and efficacy in real-world settings. Table 2 summarizes ongoing clinical trials involving Mn-based biomaterials, highlighting their diverse applications in cancer diagnosis and therapy (Table 2).

**Table 2:** Current clinical trials involving Mn-based biomaterials in cancer diagnosis and therapy

Formulations	Clinical trial phase	Clinical outcome	Advantages	ClinicalTrials.gov identifier	Ref.
Mn-DPDP (Teslascan™)	Phase III clinical trials complete	Manganese-enhanced magnetic resonance imaging of the myocardium and liver parenchyma	Enhancing the T1 signal and improving the detection of liver cancer	NCT03607669	[84]
An oral contrast containing Mn(II) (LumenHance™)	Phase III clinical trials complete	A safe and efficacious oral gastrointestinal contrast agent for MRI of the abdomen and pelvis	Reduction of the T1 relaxation time causing enhanced signal on T1 weighted MRI	Not found	[86]
Manganese chloride tetrahydrate (CMC-001, Mangoral™)	Phase III clinical trials complete	Robust liver SI enhancement in liver MRI	Significant liver signal enhancement post-oral manganese absorption with L-proline and Vitamin D3	NCT04119843	[87]
Pan-immunotherapy including MnCl_2_	Phase I clinical trial completed	Favorable clinical efficacy exhibited with 45.5% objective response and 90.9% disease control post-additional Mn^2+^ administration	Mn^2+^ Enhanced antitumor immunity via cGAS-STING and improved clinical immunotherapy efficacy	NCT03991559	[63]
Avasopasem manganese GC4419	Phase III clinical trial begun	Reducing the duration, incidence, and severity of severe oral mucositis in concurrent radiotherapy and cisplatin treatment for Head and Neck Cancer	Avasopasem manganese was an investigational selective dismutase mimetic radioprotector, reduced duration, incidence, and severity of severe oral mucositis	NCT02508389	[184]
Manganese Based Magnetic Resonance Imaging (MRI) Contrast Agent, RVP-001	Phase I clinical trial completed	Effectively assessessing myocardial infarction location, size, and transmurality	A potential alternative to gadolinium-based agents that are acceptable in the setting of renal insufficiency	NCT05413668	[83]
Mn plus chemotherapy and immunotherapy	Phase II clinical trial completed	Combining MnSOD inhibitors with conventional chemotherapeutic agents enhances ovarian cancer treatment effectiveness	MnSOD regulation is essential for anchorage-independent survival and metastasis in ovarian cancer	NCT03989336	[183]

## Conclusions and future perspectives

In recent decades, the rapid development of nanotechnology has led to an increasing number of clinical applications for functional nanomaterials. The complex valence states of manganese give rise to various Mn-based nanomaterials, including MnCl_2_, Mn complexes, and MnO nanoparticles, demonstrating excellent biocompatibility in cancer diagnostics. Notably, MnO_2_NPs are one of the most stable and versatile nanomaterials, possess important biological and immune-regulating activities. MnO_2_ nanosheets, especially, exhibit considerable potential in multimodal imaging, biosensors, and cancer therapy owing to their ultrathin structure, large surface area, high NIR absorbance, and excellent biocompatibility. The presence of vacancies in these 2D nanosheets contributes to high photothermal conversion efficiency, making them suitable for PTT to inhibit tumor growth.

Biogenic materials sourced from Mn can be used for in situ and multimodal imaging, gene/protein/drug delivery, and immunomodulation. Moreover, they serve as ideal biodegradable carriers for specific drug delivery while generating Mn^2+^ ions. Mn ions can induce the generation of hydroxyl radicals (•OH) via reactions with H_2_O_2_ in the TME. These biogenic materials catalyze Fenton-like reactions, promoting the production of highly active hydroxyl radicals (•OH) for enhanced CDT. Mn-based materials have the potential to function as biocompatible drug delivery systems that control the release of various therapeutic agents. Furthermore, apart from gadolinium CAs, MnO_2_ is one of the best alternatives for T1-weighted MRI. MnO_2_ NPs synthesized enzymatically also exhibit PTT capabilities owing to their exceptional photothermal conversion efficiency. MnO_2_ NPs, as TME-responsive drug carriers, rapidly degrade in acidic and reducing environments, enabling on-demand drug release at tumor sites. This highlights the considerable potential of Mn-based materials in cancer diagnosis, treatment, and vaccine development. Concurrently, nanodiagnostic technologies are flourishing and multifunctional nanoplatforms that combine diagnostics and treatments are crucial in future medicine.

Therefore, Mn-based nanomaterials offer the following functions in cancer diagnosis and treatment: (1) Generation of Mn^2+^ for enhanced MRI and serving as PAI and PTI CAs. (2) Acting as biocompatible nanocarriers for the delivery of immunotherapeutic and chemotherapeutic drugs to activate host anti-tumor treatments. (3) Regulation of the TME as an adjuvant to enhance immune responses. (4) cGAS-STING pathway activation to trigger tumor immunotherapy. (5) Real-time monitoring of tumor treatment effectiveness using MRI to guide PDT, CDT, and other treatments.

In this review, we focused primarily on the application of inorganic Mn-based materials for cancer diagnosis and treatment. We summarized the application mechanisms and strategies of Mn-based materials to improve the prognosis and efficacy of traditional cancer treatments, including drug delivery, TME regulation, enhanced PDT, SDT, CDT, combination therapy, and image-guided therapy. Although many researchers have developed efficient Mn-based materials for cancer treatment and diagnosis, their clinical application remains challenging. The most major limitation of manganese-based biomaterials in biological systems relates to their biocompatibility and toxicity. Although many studies suggested the good biocompatibility and low toxicity of manganese-based nanomaterials, the assessment was restricted to in vitro cell experiments. Mn-induced neurotoxicity remains a significant limit to its clinical translation, posing serious concerns for its use in medical applications. The biocompatibility of manganese-derived biomaterials, influenced by numerous factors such as size, shape, and surface charge, varies considerably. Currently, our knowledge of how nanomaterials interact with biological systems remains incomplete. Standardized methods for evaluating in vivo biocompatibility and toxicity are yet to be established. Moreover, there is considerable debate regarding how various properties of nanomaterials influence their toxicity and safety. Despite rapid advancements in nanotechnology, these issues underscore the need for further research to obtain more precise data on the potential risks and hazards associated with the biomedical applications of manganese-based nanomaterials.

Another very important limitation relates to production and application. The preparation processes for manganese-derived materials currently lack standardized protocols, leading to increased production costs and reduced overall efficiency. The absence of standardized procedures poses challenges in achieving reproducibility and scalability in the synthesis of these materials. Additionally, the complex and varied nature of manganese-derived biomaterials makes it difficult to establish a universal and cost-effective synthesis method. The need for optimization in each case contributes to higher production costs and lowers the overall efficiency of the manufacturing process. Addressing these limitations requires further research to develop standardized protocols that can enhance reproducibility, reduce costs, and improve the overall efficiency of manganese-derived biomaterials synthesis.

The synthesis of Mn-derived biomaterials is currently at the laboratory research stage, highlighting a critical demand for large-scale manufacturing of high-quality Mn-based materials. Although literature reports suggest that gram-scale synthesis of Mn-based nanoparticles is feasible through various chemical synthesis methods, achieving industrial-scale production remains unrealized. Challenges in incorporating other functional components such as nanoparticles, biomolecules, and anticancer drugs further complicate large-scale production. Therefore, the development of a simple, reliable, and scalable method for producing Mn-based nanosystems is crucial for future practical applications.

## Data Availability

No datasets were generated or analysed during the current study.

## Abbreviations

ACT: Adoptive T-cell therapy
ALV-J: Avian leukosis virus subgroup J
ASC: Alanine-serine-cysteine
ASCT2: Alanine-serine-cysteine amino acid transporter 2
amvMn: Amorphous mixed-valence manganese
ATO: Arsenic trioxide
BPNS: Black phosphorus nanosheets
CA: Contrast agent
CAR-T: Chimeric antigen receptor T-cell therapy
CDT: Chemodynamic therapy
CPIs: Checkpoint inhibitors
DDSs: Drug delivery systems
DHODH: Dihydroorotate dehydrogenase
DOX: Doxorubicin
FA: Folate
FLI: Fluorescence imaging
GBCAs: Gadolinium-based contrast agents
AuNRs: Gold nanorods
GS: Glutamine synthetase
GSH: Glutathione
HPDA: Hollow polydopamine
ICD: Immunogenic cell death
ICG: Indocyanine green
IFN: Interferons
IMD: IO@MnO_2_@DOX
LDH: Layered double hydroxide
MMn: Manganese texaphyrin
Mn SOD: Mn superoxide dismutase
Mn: Manganese
MnAs: Manganese arsenide
Mn-BGCs: Manganese-ion-doped bioactive glass-ceramic scaffolds
Mn-LDH: Manganese-based layered double hydroxide
MnO_2_NPs: MnO_2_ nanoparticles
MnP: Manganese-protoporphyrin
MRI: Magnetic resonance imaging
MS: Mesoporous silica
nanoMnSor: Manganese dioxide nanoparticles containing sorafenib
OMVs: Outer membrane vesicles
PAA: Poly (acrylic acid)
PAI: Photoacoustic imaging
PDDA: Poly (diallyldimethylammonium chloride)
PDT: Photodynamic therapy
PEC: Photoelectrochemical
PS: Mesoporous silica
PTI: Photothermal imaging
rGO: Reduced graphene oxide
ROS: Reactive oxygen species
SDT: Sonodynamic therapy
TA: Tannic acid
TME: Tumor microenvironment
UC: Upconversion
